# Meta-reinforcement learning via orbitofrontal cortex

**DOI:** 10.1038/s41593-023-01485-3

**Published:** 2023-11-13

**Authors:** Ryoma Hattori, Nathan G. Hedrick, Anant Jain, Shuqi Chen, Hanjia You, Mariko Hattori, Jun-Hyeok Choi, Byung Kook Lim, Ryohei Yasuda, Takaki Komiyama

**Affiliations:** 1 Department of Neurobiology, University of California San Diego, La Jolla, CA USA; 2 Center for Neural Circuits and Behavior, University of California San Diego, La Jolla, CA USA; 3 Department of Neurosciences, University of California San Diego, La Jolla, CA USA; 4 Halıcıoğlu Data Science Institute, University of California San Diego, La Jolla, CA USA; 5 Max Planck Florida Institute for Neuroscience, Jupiter, FL USA; 6 Present Address: Department of Neuroscience, The Herbert Wertheim UF Scripps Institute for Biomedical Innovation & Technology, University of Florida, Jupiter, FL USA

**Keywords:** Neural circuits, Reward, Cortex

## Abstract

The meta-reinforcement learning (meta-RL) framework, which involves RL over multiple timescales, has been successful in training deep RL models that generalize to new environments. It has been hypothesized that the prefrontal cortex may mediate meta-RL in the brain, but the evidence is scarce. Here we show that the orbitofrontal cortex (OFC) mediates meta-RL. We trained mice and deep RL models on a probabilistic reversal learning task across sessions during which they improved their trial-by-trial RL policy through meta-learning. Ca^2+^/calmodulin-dependent protein kinase II-dependent synaptic plasticity in OFC was necessary for this meta-learning but not for the within-session trial-by-trial RL in experts. After meta-learning, OFC activity robustly encoded value signals, and OFC inactivation impaired the RL behaviors. Longitudinal tracking of OFC activity revealed that meta-learning gradually shapes population value coding to guide the ongoing behavioral policy. Our results indicate that two distinct RL algorithms with distinct neural mechanisms and timescales coexist in OFC to support adaptive decision-making.

## Abstract (web summary)

The authors show that neural activity and synaptic plasticity in the orbitofrontal cortex mediate multiple timescales of reinforcement learning (RL) for meta-RL, which parallels a form of meta-RL in artificial intelligence.

## Main

The concept of meta-learning originates from Harlow’s psychological observation of ‘learning to learn’ in 1949 (ref. ^1^). When we learn new skills or learn to solve a new task, we do not learn each of them independently from scratch. Instead, we learn generalized knowledge through lifelong experiences in related conditions and use the knowledge to acquire new skills quickly. For example, if you have previously learned some programming languages, you can learn a new programming language more quickly using the generalized knowledge from meta-learning. Adoption of the meta-learning concept has been successful in the field of artificial intelligence (AI), allowing deep learning models to improve their own learning algorithms over multiple learning episodes^2^.

Meta-learning also applies to reinforcement learning (RL), and we often perform multiple RLs in parallel in our daily lives. Meta-RL is a meta-learning framework with distinct RL algorithms that run in parallel at distinct timescales. Deep RL models with the meta-RL framework perform multiple RLs in parallel at distinct timescales. An example implementation of meta-RL in AI uses parallel mechanisms involving synaptic plasticity and recurrent activity dynamics. In this example, a recurrent neural network performs a slow RL using its synaptic plasticity mechanism and performs another RL at a faster timescale using its recurrent activity dynamics^3–6^. Here a slow, plasticity-based RL algorithm shapes the network connectivity that gives rise to a new, faster RL that relies on recurrent activity dynamics. Previous network simulations lead to the hypothesis that the prefrontal cortex may mediate meta-RL in the brain^5^, but it is unknown whether and how plasticity- and activity-based mechanisms work together to mediate meta-RL in the brain.

In the current study, we investigated the neural mechanism of meta-RL in the mouse brain using an RL task we previously established^7,8^. We found that both mice and deep RL models perform meta-RL by a slow-timescale RL during training across sessions that gradually optimizes their behavioral action policies for a fast-timescale RL. This slow RL was mediated by Ca^2+^/calmodulin-dependent protein kinase II (CaMKII)-dependent synaptic plasticity in the orbitofrontal cortex (OFC) in the mouse brain. Following the slow RL, the neural activity in both OFC and deep RL models robustly encoded action value signals that are necessary for the fast RL behaviors. Longitudinal imaging of neural activity revealed the dynamics and stabilization of the activity-based fast RL through the plasticity-based slow RL in OFC. Although the precise learning algorithms for synaptic plasticity may differ between the mouse OFC and the deep RL models (for example, nonbiological backpropagation algorithm), both exploit a shared neural network that performs synaptic plasticity-based computations and activity dynamics-based computations for two layers of RLs. These results highlight OFC as a critical prefrontal area that mediates multiple layers of RLs in parallel to mediate meta-RL.

## Results

### Meta-learning of RL

**Fig. 1:**
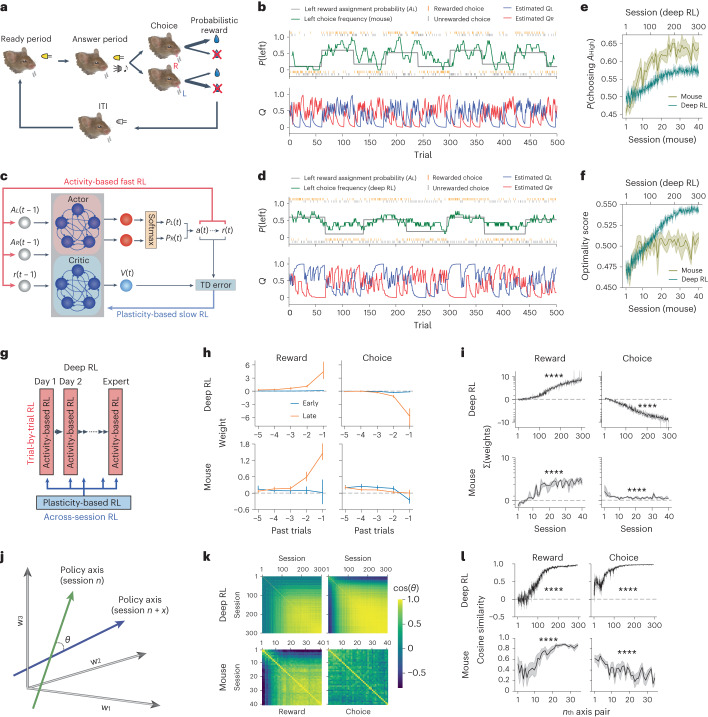
Meta-learning of RL. **a**, Schematic of the behavior task for mice. **b**, Example mouse behavior in an expert session (top) and the estimated left and right action values from an RL model in each trial (bottom). Choice frequency was calculated using nine-trial sliding windows. **c**, Schematic of the deep RL that implements meta-RL. **d**, Example behavior of a trained deep RL. **e**, Mean probability of choosing the side with a higher reward assignment probability. Note that the reward assignment probability is not equal to the reward probability in individual trials because a reward, once assigned, remains available until consumed. **f**, Mean optimality score that measures the optimality of action policy in this task considering the cumulative nature of reward availability. **g**, Schematic illustrating the meta-RL mechanism in the deep RL. Deep RL updates action values on each trial using recurrent activity, and the action policy (that is, the way they compute the values) is gradually updated by synaptic plasticity across sessions based on the performance evaluation on each session. **h**, Mean history regression weights in early (deep RL, ≤100th; OFC, day 1–14) and late (deep RL, ≥230th; OFC, ≥day 15) sessions. Mean weight was calculated using the early or late sessions for each individual, and the mean ± 95% CI of the means across models/mice is shown. **i**, Sum of the history weights of the five past trials (median ± s.e.). Both mice and deep RL models learned to use reward history for decision-making. Weights are plotted along a symmetric log scale where only the range between the minor ticks closest to 0 is linear scale (‘symlog’ option in matplotlib in Python). Deep RL (reward, *P* < 1 × 10^−100^; choice, *P* < 1 × 10^−100^), mouse (reward, *P* = 5.01 × 10^−45^; choice, *P* = 1.00 × 10^−7^). **j**, Angle between policy axes from different sessions was measured to quantify the similarity of action policies. **k**, Cosine similarity of policy axes between different pairs of training sessions. **l**, Cosine similarity between the policy axis on the *n*th session and the mean policy axis of the following 5 d (*n* + 1 − *n* + 5). Deep RL (reward, *P* < 1 × 10^−100^; choice, *P* < 1 × 10^−100^), mouse (reward, *P* = 4.1 × 10^−21^; choice, *P* = 2.58 × 10^−5^). Shadings and error bars indicate s.e. and 95% CI, respectively. Statistics in **i** and **l** are from mixed-effects models (session number as the fixed effect, subjects as the random intercept, two-sided test). NS *P* > 0.05, ****P < 0.0001. Five independently trained deep RL models and seven mice used for OFC imaging are included in **e**, **f**, **h**, **i**, **k**, and **l**. NS, not significant. Source data

**Extended Data Fig. 1:**
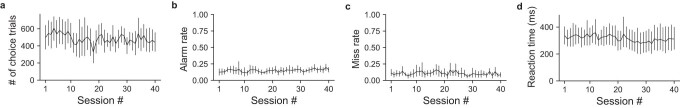
Task engagement was consistent in mice across training sessions. **a**, Mean number of choice trials per session. **b**, Mean frequency of alarm trials (licking during ready period). **c**, Mean frequency of miss trials (trials where mice did not make a choice during the 2 sec answer period). **d**, Reaction time in choice trials (across-animal averaging of median reaction time). All error bars indicate 95% CI. Only the 7 mice used for OFC imaging were included. Source data

**Extended Data Fig. 2:**
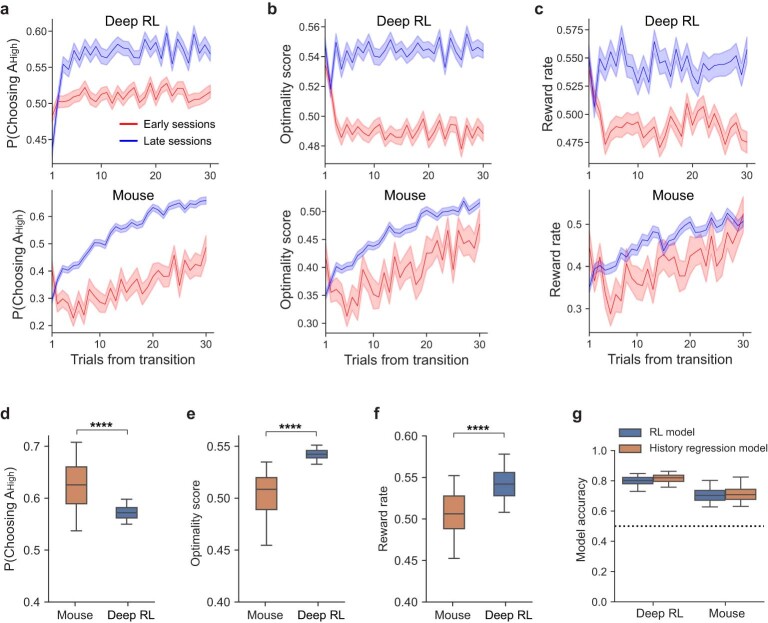
Choice adaptability after probability block transition, and the performance comparison between mice and deep RL models. **a**, Mean probability of choosing the side with a higher reward assignment probability after block transition (Deep RL: ≤100^th^ session for early and 230^th^−301^st^ sessions for late; Mouse: ≤ 5^th^ session for early and ≥15^th^ session for late). Shadings indicate s.e.m. **b**, Mean optimality score after block transition. **c**, Mean reward rate after block transition. This reward rate is mere a noisier measure of (**b**) due to the probabilistic nature of the reward assignment. Therefore, we used the quantities of (**a**) and (**b**) for most task performance quantifications. **d**–**f**, Performance comparisons between deep RL (230^th^–301^st^ sessions) and mice (≥15^th^ session). Two-sided Wilcoxon rank-sum test. The box shows the quartiles, and the whiskers extend to 5^th^ and 95^th^ percentiles. Mice developed an action policy to preferentially select the side with higher reward assignment probability, while deep RL outperformed mice by utilizing the cumulative nature of the reward availability on the unchosen side as reflected on the optimality score. This action policy difference is reflected on their choice history dependence (see Fig. 1h for choice alternation only in deep RL). **g**, Choice prediction accuracy by RL model and history regression model for expert sessions (Deep RL: ≥ 230^th^ session, mice: ≥ 15^th^ session). The RL model predicts choices as well as the regression model despite fewer parameters for both mice and deep RL models. The box shows the quartiles, and the whiskers extend to 5^th^ and 95^th^ percentiles. Data from 5 independently trained deep RL models and 7 mice used for OFC imaging are used for a-g. The ≥ 15^th^ session group of the 7 mice consisted of 292 sessions in total. ****P < 0.0001. Source data

Following pretraining in which mice were familiarized with the task apparatus and licking to receive rewards (Methods), we trained mice to learn to perform RL on a probabilistic reversal learning task^7–11^ (Fig. 1a). Head-fixed mice reported their choices with directional licking (left or right) after a ready period (2–2.5 s). On each trial, each lickport was loaded to release a water reward upon licking according to its current reward assignment probability, and mice received a reward only when the chosen lickport was loaded with a reward on the trial. Once a lickport was loaded, it remained loaded in subsequent trials until the reward was collected (baiting). The reward assignment probabilities (*A*_*L*_ and *A*_*R*_) on the lickports (0.6 versus 0.1 or 0.525 versus 0.175) changed every 60–80 trials without cue, encouraging mice to dynamically update their subjective action values for left and right based on their choice and reward outcome on each trial. Across the training sessions, mice improved their task performance by learning to adjust their choice preference dynamically toward the side that was more frequently rewarded in the recent trials (Fig. 1b and Extended Data Figs. 1 and 2a–c). We quantified their task performance by the following two measures: the probability of choosing the side with higher reward assignment probability (*P*(choosing *A*_High_)), and the average probability of reward availability on the chosen side in our task with the baiting rule (optimality score; Methods). Learning resulted in an improvement of both performance measures (Fig. 1e,f and Extended Data Fig. 2a–c). Thus, with training over sessions, mice improved their trial-by-trial RL action policy through across-session RL (that is, meta-learning of RL).

As a conceptual framework for such meta-RL, we adopted an in silico network model under the meta-RL framework^3–5^. In this framework, we trained deep RL models consisting of recurrent networks with advantage actor-critic (A2C) method^12^ (Fig. 1c). The recurrent layer receives inputs of the choice and outcome information only at the single time step immediately after a choice and incorporates them in the activity through their recurrent connectivity. Therefore, the ongoing network activity reflects the cumulative history from past trials. The action probability on each trial is computed from the network activity. The synaptic weights within the deep RL model are fixed during each training session (500 trials). However, at the end of each training session, the recurrent network updates its synaptic weights using the outer-loop RL of the meta-RL framework that evaluates the performance in each training session as a critic by calculating temporal difference (TD) errors^13^. Because of this infrequent weight updating, the networks cannot use weight updates to mediate trial-by-trial RL. In these situations, recurrent networks are encouraged to learn to use activity dynamics for the trial-by-trial RL^3–6^. While adopting this framework from previous publications^3–5^, we used recurrent networks with regular recurrent units instead of long short-term memory (LSTM) units used in other studies. LSTM units have internal gate functions that process memory signals by themselves unlike biological neurons, but in our implementation with regular recurrent units, our networks only process and maintain history signals through recurrent activity dynamics. However, we also note that the deep RL model is not intended to be an accurate network model for the brain. Instead, our goal here is to compare and contrast between the brain and the deep RL model. These deep RL models were previously used to propose the theory that a single network can perform multiple layers of RLs using synaptic plasticity- and activity dynamics-based computations^3–6^, but it has not been determined whether the brain uses similar processes to use distinct RL mechanisms in a single area to mediate meta-RL.

We trained the deep RL models in the same probabilistic reversal learning task using the meta-RL framework. As with the trained mice, the trained deep RL models dynamically adjusted their choice preference based on their choice and reward outcomes on each trial (Fig. 1d–f and Extended Data Fig. 2a–c). Notably, because the synaptic weights were fixed within each session, the results indicate that across-session plasticity established recurrent connectivity that can implement trial-by-trial RL using recurrent activity dynamics (Fig. 1g), similar to previous reports^3–5^.

Having established that both mice and deep RL models improve their task performance over training, we next examined their action policies of trial-by-trial RL during training. We quantified their history-based action policies using a logistic regression model fit to the behavior in each session. We found that both mice and deep RL models learned to choose the side that was more frequently rewarded (positive weights for reward history) in the recent trials (Fig. 1h,i). Additionally, deep RL models developed reward-independent choice alternation (negative weights for choice history) during training. This tendency for choice alternation by deep RL models is beneficial in this task because the probability of a lickport loaded with a reward cumulatively increases if the side has not been selected in recent trials^11^. Mice do not appear to make use of this feature of the task in our particular experimental condition as indicated by the nonnegative weights for choice history. As a result, mice primarily learned to choose the side with the higher predetermined reward assignment probability for the trial block (0.6 or 0.525), while deep RL models learned to exploit the cumulative nature of the reward probability and choose the side that is more likely to give reward in individual trials (Fig. 1e,f and Extended Data Fig. 2a–f). Despite this difference in the reward-independent choice effects, the overall behavior of both mice and deep RL models could be well fit by RL models (Extended Data Fig. 2g), which allowed us to estimate their subjective action values on each trial (Fig. 1b,d).

Although the summed history weights of the regression model revealed a gradual increase in the magnitude of history dependence (Fig. 1i), the analysis does not distinguish whether they changed the way they integrate history events (shape of history kernels) during training. To quantify the stability of their action policy, we defined the action policy axis for each type of history using the history regression coefficients and measured the angle between the policy axes from different sessions (Fig. 1j). We found that the reward history angle between adjacent sessions was initially large and gradually decreased, (Fig. 1k,l). Thus, both mice and deep RL models dynamically updated their reward-based action policies in early sessions and gradually stabilized their RL policies during training. Additionally, deep RL models that learned to use choice history stabilized their choice-based action policies.

These results demonstrate that both mice and deep RL models meta-learned to perform RL (that is, meta-RL) and equip us with a behavioral model that allows us to estimate the subjective action values on a trial-by-trial basis.

### OFC plasticity is required for across-session meta-learning of RL

**Fig. 2:**
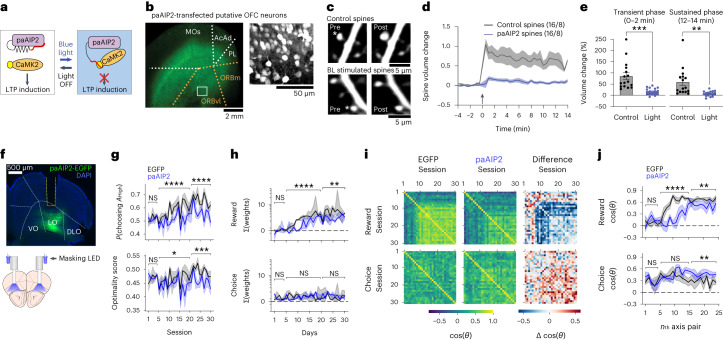
OFC plasticity is required for across-session meta-learning of RL. **a**, Schematics of optogenetic suppression of synaptic plasticity with paAIP2. **b**, Virally transfected neurons expressing mEGFP and paAIP2 in a cortical organotypic slice. Right, a field-of-view from lateral orbitofrontal cortex showing transfected pyramidal neurons. **c**, Top, representative control paAIP2-labeled dendritic shaft of the OFC neuron in which LTP induction using two-photon uncaging without paAIP2 stimulation showed an increase in the spine volume. Bottom, a representative dendritic shaft of the OFC neuron expressing mEGFP and paAIP2 in which LTP induction during blue light stimulation did not show any structural change. Fluorescence intensity of mEGFP was used to measure the spine volume change. For structural long-term potentiation (sLTP) experiments, we transfected slices nine independent times from which we recorded 16 cells in each condition. We obtained similar results as represented in **b** and **c** for these nine independent slices. **d**, Average (mean ± s.e.m.) time course summary of all spines from paAIP2-labeled OFC neurons where LTP was induced successfully without light (gray, 16 spines from 8 neurons) but failed when stimulated with light (blue, 16 spines from 8 neurons). **e**, Bar graphs showing mean transient volume change (volume change averaged over 0–2 min (mean ± s.e.m.), unpaired *t* test, *t*(30) = 4.17, *P* = 0.0002) and sustained volume change (volume change averaged over 12–14 min (mean ± s.e.m.), unpaired *t* test, *t*(30) = 3.252, *P* = 0.0028). Asterisk denote statistical significance. **f**, Histology image showing paAIP2 expression and fiber-optic cannula targeting the lateral OFC (LO). Yellow dotted line indicates the location of cannula. We confirmed that all mice in paAIP2 groups (5 mice in Fig. 2 and 5 mice in Fig. 3) in this study show similar expression patterns as in this example. **g**, Mean probability of choosing the side with a higher reward assignment probability (early, *P* = 0.95; middle, *P* = 1.89 × 10^−5^; late, *P* = 5.36 × 10^−6^), and the optimality score (early, *P* = 0.66; middle, *P* = 1.38 × 10^−2^; late, *P* = 2.74 × 10^−4^). Mice with EGFP (black, five mice) or EGFP-P2A-paAIP2 (blue, five mice) virus injections. **h**, Summed history weights (medians) across training sessions. Compared separately for days 1–5, 6–20 and 21–30. Suppression of OFC plasticity during training impairs the learning of reward-based action policy. Reward (early, *P* = 0.41; middle, *P* = 1.58 × 10^−8^; late, *P* = 2.37 × 10^−3^), choice (early, *P* = 0.87; middle, *P* = 0.65; late, *P* = 0.27). **i**, Mean cosine similarity of policy axes between pairs of training sessions and its difference between control and paAIP2 mice. **j**, Mean cosine similarity between the policy axis on the *n*th session and the mean policy axis of the following 5 d. Reward (early, *P* = 0.43; middle, *P* = 1.08 × 10^−9^; late, *P* = 2.50 × 10^−3^), choice (early, *P* = 0.80; middle, *P* = 0.20; late, *P* = 1.51 × 10^−3^). Shadings and error bars indicate s.e. and 95% CI, respectively. Statistics in **g**, **h** and **j** are from mixed-effects models (session number as the fixed effect, subject as the random intercept). Aligned rank transform for **h**. All tests are two-sided. NS *P* > 0.05, *P < 0.05 , **P < 0.01, ***P < 0.001, ****P < 0.0001. DLO, dorsolateral OFC; VO, ventral OFC. Source data

**Extended Data Fig. 3:**
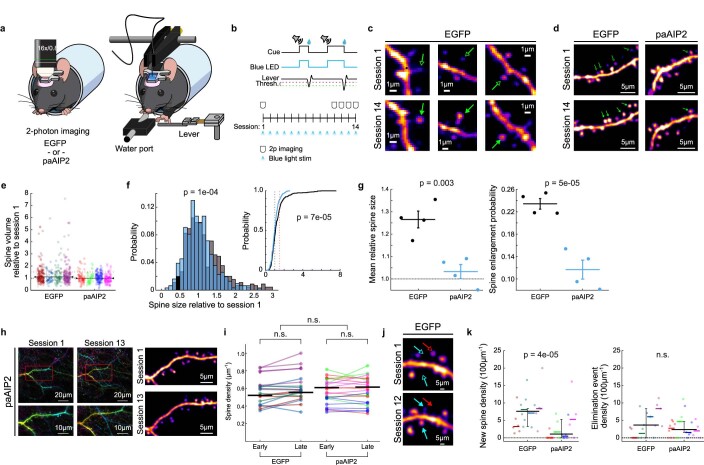
CaMKII inhibition by paAIP2 blocks dendritic spine plasticity in M1 during motor learning *in vivo* without affecting the spine density and dendritic structure. **a**, Schematic of behavior and imaging setup. Left, mice expressing either EGFP or both EGFP and paAIP2 were subjected to 2-photon imaging prior to behavioral training. Right, during training, blue light was directed into the cranial window. **b**, Top, behavioral paradigm. An auditory cue is presented, after which the lever must be pressed past both the smaller (red dotted line) and larger (green dotted line) thresholds in order to receive a water reward. Blue light is on during all cue periods. Bottom, longitudinal experimental schedule. Imaging was performed prior to behavior on sessions 1, 11, 12, 13, and 14. For each field of view, of the 11^th^-14^th^ session, one with the best image quality was chosen and used as the late session. Blue light was presented during every session. **c**, Selected examples of spine enlargement in EGFP control animals. Unfilled arrowheads demarcate the spines of interest prior to enlargement. Filled arrowheads indicate the spines after enlargement in late imaging sessions. **d**, Example images illustrating the prevalence of spine enlargement along dendrites in EGFP controls and paAIP2-expressing mice. Demarcated spines are those showing >= 1.5× volume relative to session 1. Filled and unfilled arrows demarcate spines before and after enlargement, respectively. The probability of spine enlargement shown here is comparable to the average values reported in **g**. **e**, Spine volume measurements from late sessions of training relative to the first session of training. Data points correspond to individual spines. Only spines present in both early and late sessions (‘stable spines’) are shown. Colors represent individual animals. The median value of each animal (color-coded horizontal bars) as well as the median of these values for each group (black bars with centripetal arrowheads) are shown. Black dotted line corresponds to a relative spine volume of 1, indicating stable spine size over the experiment. Red dotted line indicates the spine enlargement threshold (1.5× session 1 size) used in subsequent analyses. n = 449 stable spines / 25 dendritic segments / 5 neurons / 4 mice for EGFP controls, n = 308 stable spines, 18 dendritic segments / 5 neurons / 4 mice for paAIP2. **f**, Histograms of changes in spine size over motor learning for EGFP- (gray) and paAIP2 (light blue)- expressing mice. Both groups show a primary peak at 1, indicating that a majority of spines are relatively stable in their size. The median relative spine size in EGFP controls (1.08, 95% CI = [1.04 1.11]) is nonetheless higher than for paAIP2-expressing mice (0.98, 95% CI = [0.93, 1.03]; p = 1e-04, rank-sum test). A pronounced upper tail is apparent in the EGFP distribution. Inset, corresponding cumulative data distributions for EGFP- (black) and paAIP2- (light blue) expressing mice. The distributions are significantly different (p = 7e-05, Kolmogorov-Smirnoff test), and the lower representation of spine enlargement (>1.5×, red dotted line) in the paAIP2 animals is apparent. Statistical tests are two-sided **g**, Summary of motor learning-related changes in spine size by animal. Left, mean changes in spine size are reduced in paAIP2-expressing animals (p = 0.003, two-sample t-test). Data points correspond to the mean of all measured spines for each animal. n = 449 stable spines / 25 dendritic segments / 5 neurons / 4 mice for EGFP controls, n = 308 stable spines, 18 dendritic segments / 5 neurons / 4 mice for paAIP2. The means of animals for each group are plotted as color-coded horizontal bars. Error bars correspond to mean ± SEM across animals. Right, the probability of spine enlargement (> 1.5×) is significantly lower in paAIP2-expressing animals (p = 5e-05, chi-square test of proportions). Mean ± SEM. Note that the nonzero enlargement probability in paAIP2 animals indicates that plasticity is still occurring, albeit at a lowered level. Statistical tests are two-sided. **h**, Example *in vivo* images illustrating the viability of paAIP2-expressing neurons across multiple imaging sessions. Left, example *in vivo* images of a dendrite in early and late imaging sessions. Zoomed-in versions of the selected dendritic segment (red box) on both sessions are shown at bottom. Right, an extracted portion of the dendrite demarcated at left. The majority of spines are stable, and there is no apparent sign of diminishing dendritic health. Images are shown as color-coded by depth to illustrate out-of-plane structures. **i**, Overall spine density is comparable between EGFP- and paAIP2-expressing mice, and is stable over time. Individual dendritic segments used in this analysis are shown as partially transparent points/lines for both early and late sessions, color-coded by animal. The median spine density for each animal is plotted as color-matched opaque lines. The medians across animals are plotted as black lines. There is no main effect of training session (that is early vs. late, p = 0.47; 2-way ANOVA) nor of transgene (that is EGFP vs. paAIP2, p = 0.38; 2-way ANOVA) on spine density. Further, there is no significant interaction between training session and transgene. Together, these data illustrate that spine density is stable over training, irrespective of the transgene being expressed. n = 628 spines / 20 dendritic segments / 4 mice for EGFP controls; n = 614 spines / 23 dendritic segments / 4 mice for paAIP2. The mean dendritic segment length was 50 ± 7μm for EGFP and 50 ± 10μm for paAIP2. **j**, Example *in vivo* images illustrating spine turnover in EGFP control mice. Both spine formation (cyan arrows) and spine elimination (red arrows) are apparent on the dendritic segment shown. Unfilled arrows indicate the location of future formation or elimination; filled arrows indicate the corresponding state in the late learning session. **k**, Summary of spine turnover in EGFP- and paAIP2-expressing mice. Left, new spine density measured along dendritic segments (each data point represents 1 dendritic segment) from late imaging sessions for each animal (color-coded data points). The median density and 95% confidence intervals (after first taking the median of each animal) are shown in black (EGFP: median 8 new spines / 100μm, 95% CI: [3, 9]; paAIP2: median 1 new spine / 100μm, 95% CI: [0, 5]). When considering individual dendrites as a sample, EGFP-expressing dendrites show a significantly higher new spine density (p = 4e-05, rank-sum test). When considering animals as a sample, there is a trend in the same direction (p = 0.057, rank-sum test). Right, density of spine elimination events along the same dendritic segments. Elimination density is comparable between EGFP dendrites (median = 3 eliminations / 100μm) and paAIP2 dendrites (median = 2 eliminations / 100μm), showing no significant difference when considering individual dendrites (p = 0.93, rank-sum test) or animals (p = 1, rank-sum test) as samples. n = 67 new spines / 33 eliminated spines / 20 dendritic segments / 4 mice for EGFP controls; n = 19 new spines / 31 eliminated spines / 23 dendritic segments / 4 mice for paAIP2. All tests are two-sided.

In the deep RL model with the meta-RL framework, slow synaptic plasticity across sessions during training establishes a network that implements trial-by-trial RL using recurrent activity dynamics (Fig. 1g). We examined whether a similar mechanism is involved in mice. We focused on OFC as a candidate area that may mediate meta-RL in the mouse brain. Previous studies showed that OFC neurons encode value signals^14–19^ and undergo structural synaptic plasticity in reward-based learning^20–22^. Furthermore, OFC forms reciprocal connections with ventral tegmental area (VTA) neurons^23–28^, a source of TD error in the brain^29^. Based on these previous findings, we hypothesized that synaptic plasticity in OFC is involved in the slow across-session meta-learning of meta-RL in the mouse brain. To test this hypothesis, we sought to block the plasticity induction by targeting CaMKII, a master regulator of synaptic plasticity in the brain^30^. We used paAIP2, a light-inducible inhibitor of CaMKII kinase activity^31–33^ that can block synaptic plasticity and impair learning^31,33^ (Fig. 2a). Notably, photoactivated paAIP2 selectively blocks the induction of long-term potentiation (LTP) without affecting the CaMKII function of LTP maintenance^31,32^, leaving the connectivity established before the photoactivation intact. We performed experiments in OFC slices and confirmed that photoactivation of paAIP2 potently blocks structural LTP in dendritic spines (Fig. 2b–e). Furthermore, we validated its in vivo efficacy in the mouse primary motor cortex (M1) during a lever-press motor learning task, a well-established paradigm that induces synaptic plasticity^34–36^. We found that the increase of spine volume and the formation of new spines over days of learning were suppressed by photoactivation of paAIP2 in M1 neurons, and the neurons maintained their health with normal dendritic structures and spine density after 2 weeks of daily photoactivations (Extended Data Fig. 3). Although we did not measure the effect of paAIP2 on functional plasticity, structural plasticity has been repeatedly shown as an accurate proxy for functional plasticity^37,38^.

**Extended Data Fig. 4:**
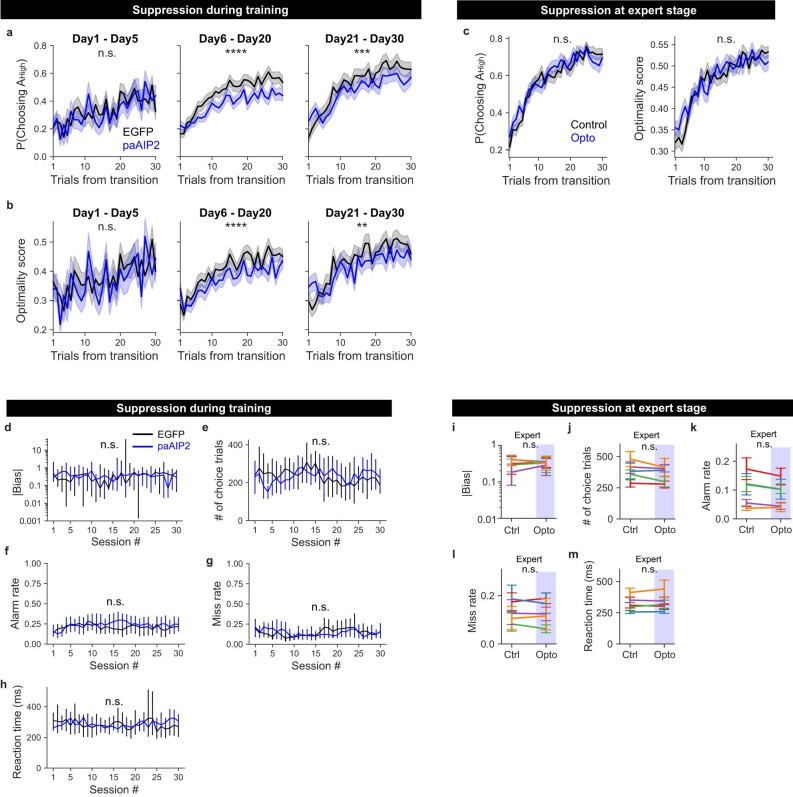
Suppression of OFC plasticity delays the improvement in the choice adaptability after each probability block transition during training, but the same manipulation in expert mice did not impair their choice adaptability. **a**, Probability of choosing the side with a higher reward assignment probability after probability block transition for different learning phases (Day 1–5: p = 0.44, Day 6–20: 2.40 × 10^–16^, Day 21–30: 1.12 × 10^−4^). **b**, Optimality score after probability block transition for different learning phases (Day 1–5: p = 0.25, Day 6–20: 1.41 × 10^−7^, Day 21–30: 5.22 × 10^−3^). **c**, The same probabilities for paAIP2-expressing mice that received photoactivation only after achieving expert performance. This mouse group is separate from the group used in (**a** and **b**). Sessions with [photoactivation + masking light] and [masking light only] were alternated for 20 sessions. Although OFC plasticity suppression delayed the across-session meta-learning, it did not affect the trial-by-trial RL of expert mice. Shadings indicate s.e.m. **d-h**, Suppression of OFC plasticity during training did not affect history-independent action bias and task engagement. **d**, Median size of history-independent action bias for mice with both EGFP and paAIP2 expression (blue) or with only EGFP expression (black). **e**, Mean number of choice trials per session. **f**, Mean frequency of alarm trials (licking during ready period). **g**, Mean frequency of miss trials (trials where mice did not make a choice during the 2 sec answer period). **h**, Reaction time in choice trials (mean of median reaction time). Statistics are from mixed effects models (virus as the fixed effect and session as the random intercept). **i-m**, Suppression of OFC plasticity at expert stage did not affect history-independent action bias and task engagement. Same metrics as (**d**-**h**) for paAIP2-expressing mice that received photoactivation only after achieving expert performance. This mouse group is separate from the group used in (**d**-**h**). Sessions with [photoactivation + masking light] and [masking light only] were alternated for 20 sessions. Each line indicates the mean per mouse (10 sessions for each condition). All error bars are 95% CI. Statistics in a-c are from mixed-effects model (suppression as the fixed effect, trials from transition as the random intercept). Statistics in **d**-**h** are from mixed-effects model (suppression as the fixed effect, session as the random intercept). Statistics in i-m are from mixed effects models (suppression as the fixed effect, subject as the random slope, session as the random intercept). All tests are two-sided. n.s. P > 0.05, **P < 0.01, ***P < 0.001, ****P < 0.0001. Source data

**Extended Data Fig. 5:**
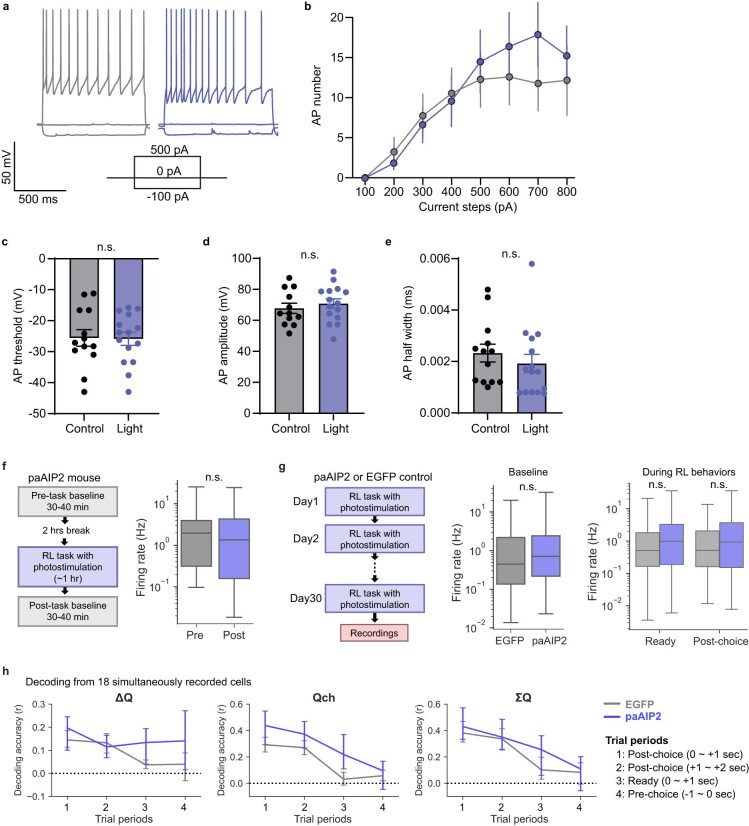
CaMKII inhibition by paAIP2 does not affect firing properties and value coding of OFC neurons. **a-e**. Recordings from OFC slices. **a**, Representative traces of whole-cell current-clamp recordings from paAIP2 labeled OFC pyramidal neurons in organotypic cortical slices at three different current steps (−100, 0, and 500 pA) before (gray) and after (blue) 40 minutes of blue light stimulation (1 sec ON, 3 sec OFF). The recordings were made from different groups of neurons before and after blue light stimulation. **b**, Mean (± SEM) number of action potentials (AP) evoked by depolarizing current steps. n = 15 cells after stimulation and 13 cells from 5 transfected slices before stimulation. **c**, Summary of AP threshold (mean ± SEM) showing no difference between before and after stimulation cells (p = 0.93, unpaired t-test, t(26) = 0.08, p = 0.92). **d**, Summary of AP amplitude (mean ± SEM) showing no difference between before and after stimulation cells (p = 0.5, unpaired t-test, t(26) = 0.68, p = 0.5). **e**, Summary of AP half-width (mean ± SEM) showing no difference between before and after stimulation cells (p = 0.42, unpaired t-test, t(26) = 0.80, p = 0.42). **f-h**, Recordings *in vivo*. **f**, Baseline firing rates of OFC neurons were recorded with a chronic silicone probe under head-fixation in darkness 2 hrs before and immediately after a behavior session with paAIP2 photoactivation. n = 25 cells for pre-task and 22 cells for post-task. The box shows the quartiles, and the whiskers extend to the minimum and maximum. Two-sided Wilcoxon rank-sum test. **g**, Baseline firing rates (head-fixation in darkness) and firing rates during the RL task after 30 consecutive photoillumination sessions for control (EGFP only) and paAIP2 mice. The firing rates during the task were calculated from the first 2-sec window of the ready or post-choice period. Baseline: n = 92 cells for EGFP and 111 cells for paAIP2. Task: n = 131 cells for EGFP (gray) and 81 cells for paAIP2 (blue). The box shows the quartiles, and the whiskers extend to the minimum and maximum. Two-sided Wilcoxon rank-sum tests. **h**, Decoding of value-related signals from the neural population activity after 30 consecutive photostimulation sessions. We used only the recorded populations with at least 18 simultaneously recorded cells, and the decoding was performed with randomly subsampled population (18 cells) to match the number of input cells to the decoder. We obtained 5 distinct populations for EGFP and 3 distinct populations for paAIP2 with at least 18 simultaneously recorded cells. The decoding analysis indicates that OFC neurons stay healthy with normal value coding in their activity after 30 consecutive paAIP2 photoactivation sessions. Error bars indicate 95% CI. Source data

To examine the involvement of OFC plasticity in across-session meta-learning of RL, we virally expressed paAIP2 locally in OFC neurons and photoactivated paAIP2 for 3 s after every choice throughout consecutive 30 training sessions (Fig. 2f). We compared their across-session learning of history-based action policy against the control group from the same litters with EGFP expression without paAIP2. We found that the task learning, acquisition of history dependence and stabilization of reward-based action policy were delayed in the paAIP2 group (Fig. 2g–j and Extended Data Fig. 4a,b). Plasticity suppression by paAIP2 did not affect task engagement or general task performance such as the choice bias (Extended Data Fig. 4d–h). We confirmed that paAIP2 photoactivation did not alter the firing properties of OFC neurons in organotypic slices (Extended Data Fig. 5a–e), and the baseline firing rates of OFC neurons were not affected by ~1 h of photoactivation during the RL task in vivo (Extended Data Fig. 5f). To examine potential long-term effects on OFC neurons, we also recorded OFC neural activity after consecutive 30 training sessions with paAIP2 photoactivations. We found that this long-term paAIP2 photoactivation did not alter firing rates at the baseline, firing rates during the RL task or population value coding (Extended Data Fig. 5g,h). Therefore, OFC neurons maintained healthy firing properties with our experimental protocol, and the selective paAIP2 effects on learning indicate that local synaptic plasticity in OFC is necessary for the efficient, across-session meta-learning of the RL action policy in mice.

### Trial-by-trial RL is independent of CaMKII-dependent synaptic plasticity in OFC

**Fig. 3:**
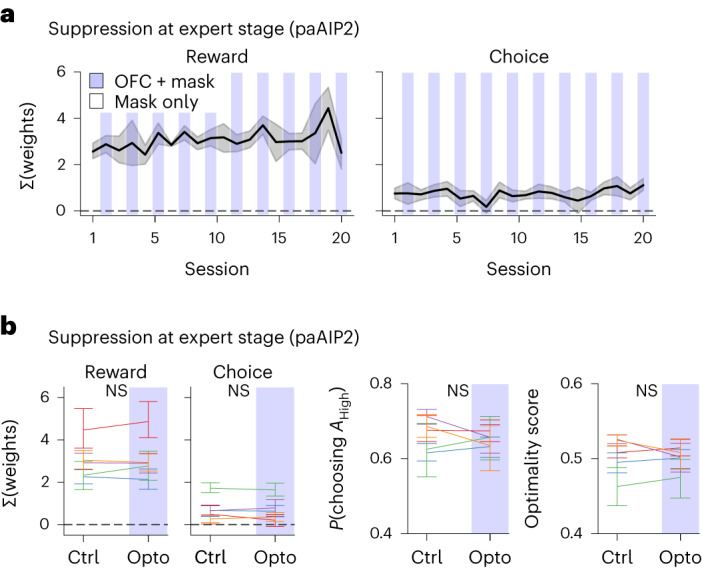
Trial-by-trial RL is independent of CaMKII-dependent synaptic plasticity in OFC. Photoactivation of paAIP2 on every other session in expert mice (five mice, blue shadings indicate photoactivation sessions). **a**, Summed history weights in individual expert sessions. **b**, Pairwise comparisons of the photoactivation effects. Each line indicates the mean per mouse. Suppression of OFC plasticity in expert mice does not affect history-based action policy and task performance. Shadings and error bars indicate s.e.m. and 95% CI, respectively. All statistics are from mixed-effects models (virus as the fixed effect, session as the random intercept, subject as the random slope, two-sided). NS *P* > 0.05. Source data

The abovementioned results are consistent with the notion that OFC plasticity during learning establishes a circuit that implements within-session, trial-by-trial RL using activity dynamics, similar to the deep RL model with the meta-RL framework. However, it is possible that OFC plasticity might also contribute to within-session RL by reflecting value updates with updates of synaptic weights. Therefore, we next tested whether OFC plasticity is necessary for within-session RL in expert mice. A cohort of paAIP2-expressing mice was first trained without photoactivation until their performance reached the expert level. We then performed paAIP2 photoactivation on alternating sessions using the same light protocol as in the learning experiments described above. To minimize nonspecific effects of light such as distraction in photoactivation sessions, another pair of light-emitting diodes (LEDs) was used to shine a masking blue light over the mouse head in every trial in both photoactivation and control sessions. Unlike the photoactivation experiments during the learning phase, blocking OFC plasticity after the acquisition of an expert action policy did not cause any detectable changes in the task performance and history dependence of mouse behavior (Fig. 3 and Extended Data Fig. 4c,i–m). Although we cannot exclude potential contributions of CaMKII-independent forms of plasticity (for example, short-term plasticity on presynaptic neurotransmitter release) that are not blocked by paAIP2, our results indicate that CaMKII-dependent plasticity in OFC is selectively required for the meta-learning of RL but not for the within-session RL of expert mice.

### OFC activity robustly encodes value signals

After the across-session RL, deep RL models perform trial-by-trial RL using their recurrent activity. Thus, we next examined whether trial-by-trial RL in expert mice is mediated by OFC activity.

**Fig. 4:**
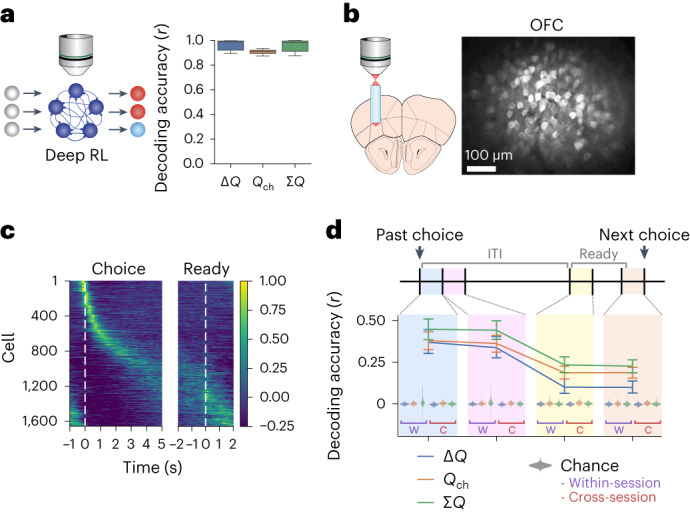
OFC activity robustly encodes value signals. **a**, Decoding accuracy of value-related signals from recurrent units of trained deep RL models (230th–301st sessions from five independently trained networks). The mean activity of the three time steps immediately before choice was used. The box shows the quartiles, and the whiskers extend to the 5th and 95th percentiles. **b**, Example calcium signals in OFC (max-intensity projection). **c**, Trial-averaged activity of OFC neurons, aligned to choice (left) or the start of the ready period (right). Cells were sorted by the peak activity timing from half of the recorded trials, and the mean activity in the other half of the trials is shown. Cells from 14 unique populations (seven mice, two planes each) were pooled. Activity of each cell was normalized to its trial-averaged peak. For each unique population, only a single expert session with the best Δ*Q* decoding accuracy at the ready period was included for this plot. **d**, Decoding accuracy of value-related signals from OFC population activity (subsampled 55 cells per population) at different trial periods (mean ± 95% CI). The updated value signals are available in OFC until the next choice. All sessions after ≥14 d of training were analyzed for all mice. To minimize spurious correlations of slowly varying neural signals and value, we decoded the change in value from change in neural activity between adjacent trials. Chance decoding accuracy was obtained by shuffling behavior labels across trials for each session (within-session) or decoding unshuffled behavior labels from different sessions (cross-session). The chance distributions are shown as kernel densities. All accuracies were significantly above chance (*P* < 1 × 10^−100^, mixed-effects model with shuffling as the fixed effect, neural population as the random intercept, two-sided). Source data

We first examined the encoding of action value-related signals, which are necessary for the trial-by-trial RL. We analyzed how the network activity encoded the following three value-related signals: Δ*Q* (value difference between left and right), which is the policy value that directly drives decision-making; ∑*Q* (sum of two action values), which reflects state value and motivation and *Q*_ch_ (value of the side chosen in the previous trial), which is the value that was updated by the preceding action. As expected, in the trained deep RL models, these value signals could be reliably decoded from the population activity in the recurrent layer on each trial at the time steps before the decision (Fig. 4a).

**Extended Data Fig. 6:**
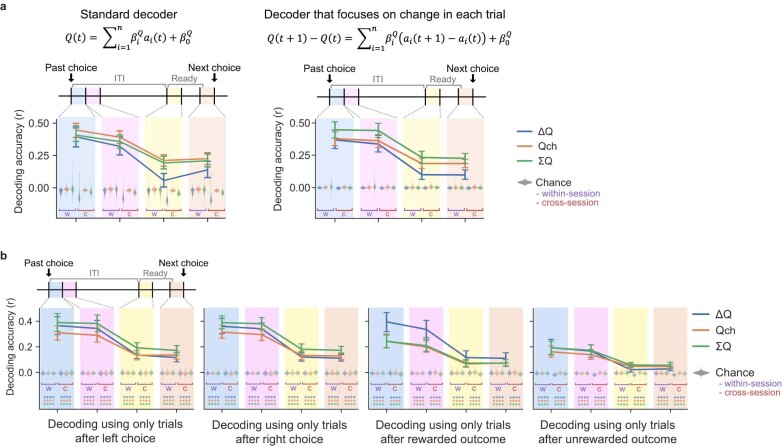
Value decoding from OFC population activity. **a**, Value-related signals were decoded from OFC population activity (subsampled 55 cells/population) with 2 different decoders (mean ± 95% CI). Standard decoder (left) directly decodes value-related signals from neural population activity on individual trials. Trial-derivative decoder (right) decodes the change in value-related signals from the change in population activity between adjacent trials (duplicate from Fig. 4d). Both decoders decoded significant value-related signals from OFC through the trial periods. All sessions after ≥ 14 days of training were analyzed for all mice. Chance decoding accuracy was obtained by shuffling behavior labels across trials for each session (‘within-session’) or decoding unshuffled behavior labels from different sessions (‘cross-session’). The chance distributions are shown as kernel densities. All accuracies were significantly above chance (P < 0.0001). **b**, Decoding accuracy of value-related signals from OFC population activity (subsampled 55 cells/population) at different trial periods when the decoder was trained and tested using either only left choice trials, right choice trials, rewarded trials, or unrewarded trials (mean ± 95% CI). All accuracies were significantly above chance (P < 0.0001). Decoding was performed with 10-fold CV. Chance decoding accuracy for each condition was obtained by shuffling the behavior labels across trials for each session (‘within-session’) or decoding unshuffled behavior labels from different sessions (‘cross-session’). The chance distributions are shown as kernel densities. These results indicate that the decoding of value-related signals from OFC is not reflecting those binary signals that may partially correlate with values (for example choice for ΔQ, and reward for ∑Q). Statistics are from mixed effects model (shuffling as the fixed effect, neural population as the random intercept, two-sided). ****P < 0.0001. Source data

To examine value coding in OFC, we performed in vivo two-photon calcium imaging of layer 5/6 OFC neurons. This was done through an implanted gradient-index (GRIN) lens in CaMKIIa-tTA::tetO-GCaMP6s double transgenic mice that express GCaMP6s in cortical excitatory neurons^7,39,40^ (Fig. 4b). Extracted fluorescence signals from individual neurons were deconvolved to estimate the underlying spiking activity before analyses (Supplementary Fig. 1)^41,42^. OFC neurons were heterogeneous with different neurons exhibiting activity peaks at different trial periods, collectively tiling the entire trial period (Fig. 4c). We examined population coding of value-related signals by decoding analyses. Because both action values and neural activity can slowly change throughout a session, they may show spurious correlations^43–46^. To minimize spurious correlations that derive from the slow-timescale autocorrelations of individual variables, we designed a decoder to decode the difference in the value-related signals from the difference in the population activity between adjacent trials. To confirm that this approach of decoding differences between adjacent trials reduces spurious correlations, we calculated the chance accuracy by shuffling variables across sessions. We found that the chance accuracy with the trial-difference decoder was close to zero, closer to zero than the chance accuracy of a standard decoder that decodes signals in individual trials (Extended Data Fig. 6a). We found that Δ*Q*, ∑*Q* and *Q*_ch_ were significantly encoded in the population activity throughout the entire trial period until the next choice (Fig. 4d). These signals could be reliably decoded even when the decoding analysis was performed using exclusively either left, right, rewarded or unrewarded trials (Extended Data Fig. 6b), indicating that the value decoding is not merely reflecting those binary signals.

### OFC activity is necessary for trial-by-trial RL

**Fig. 5:**
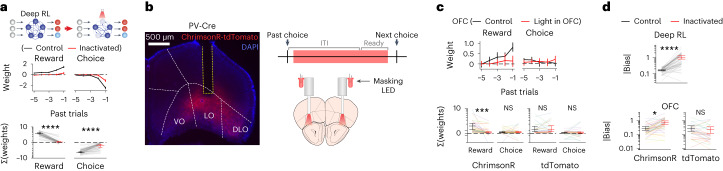
OFC activity is necessary for trial-by-trial RL. **a**, Inactivation of the recurrent activity of deep RL models at the prechoice time step impairs behavioral dependence on history (30% of cells were inactivated, *P* < 1 × 10^−10^ for both). Mean regression weights of 50 sessions (top), and the sum of each type of history weights from the past five trials (bottom). **b**, Schematics and a histology image for bilateral OFC inactivation. Inactivation was performed in ~13% of trials throughout the duration of ITI (0.5 s delay) and ready period. Yellow dotted line indicates the location of the cannula. **c**, Bilateral optogenetic inactivation of OFC impairs reward history dependence. Mean regression weights for mice with ChrimsonR-tdTomato (top), and the sum of each type of history weights from the past five trials for mice with ChrimsonR-tdTomato or only tdTomato (bottom). Different colors of thin lines indicate different mice. Black, control trials; red, light-on trials. Inactivation impairs reward history dependence (*P* = 8.01 × 10^−4^). **d**, Mean inactivation effects on the size of history-independent action bias for deep RL and mice. Black, control trials; red, inactivation trials. Inactivation increased dependence on the bias in both mice (*P* = 0.018) and deep RL (*P* < 1 × 10^−10^). All error bars are 95% CI. All statistics are from mixed-effects model with aligned rank transform (inactivation as the fixed effect, subject as the random slope, session as the random intercept for mice; inactivation as the fixed effect, session as the random intercept for deep RL). All tests are two-sided. NS *P* > 0.05, *P < 0.05, ***P < 0.001, ****P < 0.0001. ChrimsonR-tdTomato (6 mice, 43 sessions) and tdTomato (5 mice, 30 sessions) for **c** and **d**. Source data

**Extended Data Fig. 7:**
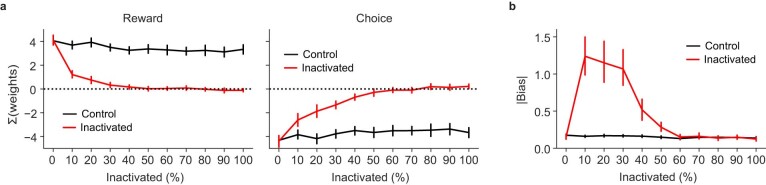
Effects of inactivation in deep RL models on behavioral action policy with different fractions of inactivated recurrent units. **a**, Sum of each type of history weights from the past 5 trials for control (black) and inactivation (red) trials (mean ± 95% CI). **b**, Size of history-independent action bias for control (black) and inactivation (red) trials (mean ± 95% CI). Different fractions of recurrent units were inactivated. For each fraction condition, neurons to be inactivated were randomly selected for each session. The random subsampling of neurons was repeated 50 times for each fraction condition. Source data

Next, we investigated the involvement of the population activity in the trained deep RL model and mouse OFC in the expert task performance. We first simulated transient inactivation of trained deep RL model by silencing the prechoice activity of a subset of neurons in the recurrent layer in ~13% of randomly interleaved trials. Figure 5a shows inactivation of 30% of neurons, while Extended Data Fig. 7 shows additional proportions of inactivated neurons. The action policies on inactivation and control trials were examined by the history regression model as above. Deep RL inactivation significantly decreased the dependence on history (Fig. 5a and Extended Data Fig. 7a).

**Extended Data Fig. 8:**
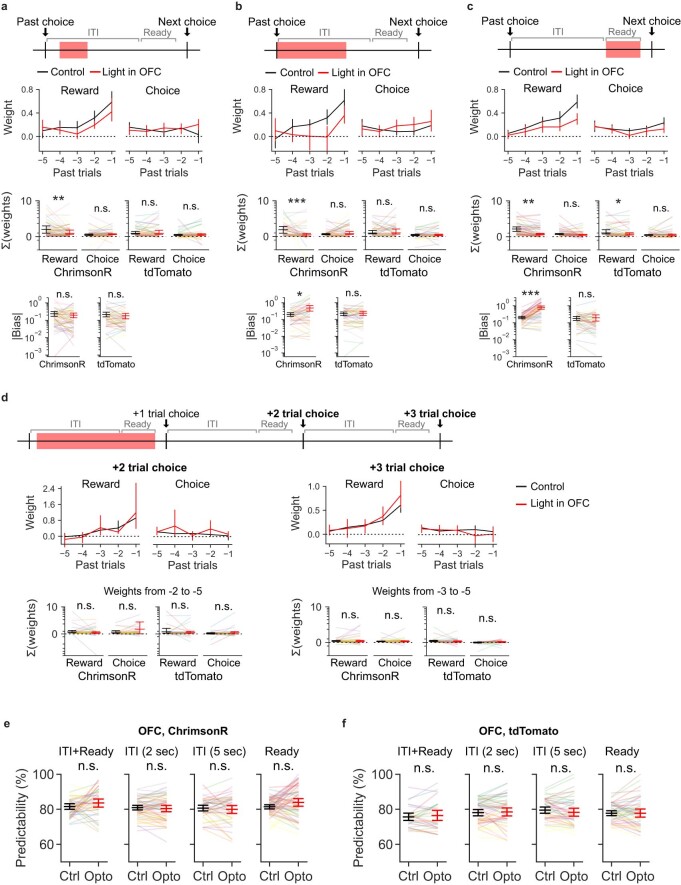
OFC inactivation during ITI or ready period impairs behavioral action policy based on reward history. **a**, Optogenetic inactivation of OFC during 2 sec ITI (1–3 sec after choice). ChrimsonR-tdTomato: 9 mice, 60 sessions. tdTomato: 8 mice, 49 sessions. [1^st^ row]: Inactivation period. [2^nd^ row]: Mean regression weights for mice with ChrimsonR-tdTomato. Black, control trials; red, inactivation trials. [3^rd^ row]: Sum of each type of history weights from the past 5 trials for mice with ChrimsonR-tdTomato or only tdTomato. Black, control trials; red, light-on trials. Different colors of thin lines indicate different mice. Horizontal bars indicate mean ± 95% CI. [4^th^ row]: Inactivation effects on the size of history-independent action bias. Horizontal bars indicate mean ± 95% CI. Different colors of thin lines indicate different mice. Inactivation impaired reward history dependence (a: p = 0.0041, b: p = 2.26 × 10^−4^, c: p = 0.0027) and |Bias| (b: p = 0.013, c: p = 6.01 × 10^−4^). **b**, Optogenetic inactivation of OFC during 5 sec ITI (0-5 sec after choice). ChrimsonR-tdTomato: 8 mice, 47 sessions. tdTomato: 8 mice, 46 sessions. **c**, Optogenetic inactivation of OFC during the ready period. ChrimsonR-tdTomato: 10 mice, 62 sessions. tdTomato: 8 mice, 49 sessions. **d**, Effects of ITI+Ready inactivation on the choices 2 or 3 trials later. Sum of history weights between -2 and -5 trials was used for the +2 trial effect comparison, and the sum of history weights between -3 and -5 trials was used for the +3 trial effect comparison. Significant inactivation effect was restricted to the immediately following trial (Fig. 5c). **e**, Fractions of mouse choices that were correctly predicted by the history-based regression model for mice with ChrimsonR-tdTomato expression. Horizontal bars indicate mean ± 95% CI. **f**, Same as (e) for mice with tdTomato expression. All error bars are 95% CI. Statistics are from mixed effects model with Aligned-Rank-Transform (inactivation as the fixed effect, subject as the random slope, session as the random intercept, two-sided). n.s. P > 0.05, *P < 0.05, **P < 0.01, ***P < 0.001. Source data

To evaluate the effect of OFC inactivation on expert mouse performance, we performed optogenetic inactivation (Fig. 5b). We injected adeno-associated virus (AAV) encoding Cre-dependent ChrimsonR^47^, a red-light-gated cation channel, in the OFC of PV (Parvalbumin) -Cre transgenic mice. Red light was delivered through fiber-optic cannulas to inactivate OFC bilaterally by activating local PV-expressing inhibitory neurons in ~13% of randomly interleaved trials. Another pair of LEDs was used to shine a red light over the mouse head in every trial as the masking light. When OFC was bilaterally inactivated throughout the ITI and ready period, behavioral dependence on reward history was largely abolished in inactivation trials (Fig. 5c). Furthermore, inactivation only during ITI or ready period also significantly reduced dependence on reward history (Extended Data Fig. 8a–c). The effects of inactivation were restricted to the decision immediately following inactivation, and the history dependence largely recovered in the following trials (Extended Data Fig. 8d). In contrast, control mice with only tdTomato expression in PV-expressing inhibitory neurons without ChrimsonR did not show any significant changes to their action policies after the same red-light delivery into the OFC.

The decreased behavioral dependence on reward history may result in either more stochastic decision-making, an increased dependence on choice history, or an increased dependence on history-independent action bias that is static within each session. We found that the predictability of their choice patterns with the regression model was not affected by inactivation in mice (Extended Data Fig. 8e,f), suggesting that inactivation did not increase the stochasticity of decisions. Instead, the history-independent idiosyncratic action biases substantially increased by inactivation in both deep RL models and mice (Fig. 5d and Extended Data Figs. 7b and 8a–c).

**Fig. 6:**
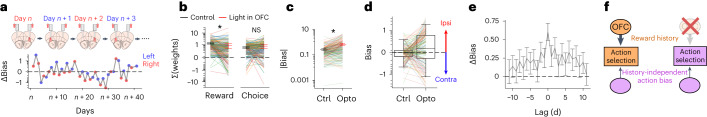
History-independent action bias is independent of OFC. **a**, Unilateral OFC inactivation, alternating the side of inactivation every session. The impact on the history-independent bias direction in an example mouse (ΔBias = (bias in inactivated trials) − (bias in control trials)) is shown. **b**, Mean effects of unilateral OFC inactivation on history dependence (4 mice, 177 sessions). Black, control trials; red, inactivation trials. Similarly to bilateral inactivation, behavioral dependence on reward history was impaired by unilateral inactivation (*P* = 0.018). **c**, Unilateral inactivation increased the size of the mean unsigned bias (*P* = 0.030). **d**, The direction of the bias did not depend on the side of unilateral inactivation. The box shows the quartiles, and the whiskers extend to the 5th and 95th percentiles. **e**, Mean bias direction across days. The sign of ΔBias was flipped for those with negative ΔBias at day 0. The direction of enhanced bias was generally consistent for several days. **f**, Action selection based on reward history requires OFC. When OFC is inactivated, history-independent action bias dictates action selection. All error bars are 95% CI. All statistics are from mixed-effects model with aligned rank transform (inactivation as the fixed effect, subject as the random slope, session as the random intercept, two-sided). NS *P* > 0.05, *P < 0.05. In total, 177 sessions from 4 mice are used for **b**–**e**. Source data

Previous studies have found that unilateral inactivation of several brain areas such as premotor cortex and striatum can lead to a lateralized choice bias toward the direction ipsilateral to the inactivated hemisphere^48,49^. Therefore, we tested whether the direction of the increased history-independent action bias depends on the side of the inactivated hemisphere. We performed unilateral OFC inactivation (ITI + ready) of expert mice with the inactivation side flipped on alternating days (Fig. 6a). Overall, the effects of unilateral inactivation were similar to bilateral inactivation, with decreased dependence on history and increased dependence on history-independent biases (Fig. 6b,c). Notably, the direction of the increased bias did not depend on the hemisphere that was inactivated (Fig. 6d). Instead, the bias direction slowly drifted across sessions irrespective of inactivated hemispheres (Fig. 6e). Thus, the OFC does not appear to be directly controlling history-independent decision biases. Rather, our results suggest that the OFC is selectively involved in history-dependent value-based decision-making (Fig. 6f). When OFC is inactivated, the action selection circuit relies mostly on the history-independent bias that is independent of OFC and slowly drifts over days.

### Dynamics and stabilization of value coding during meta-learning

**Fig. 7:**
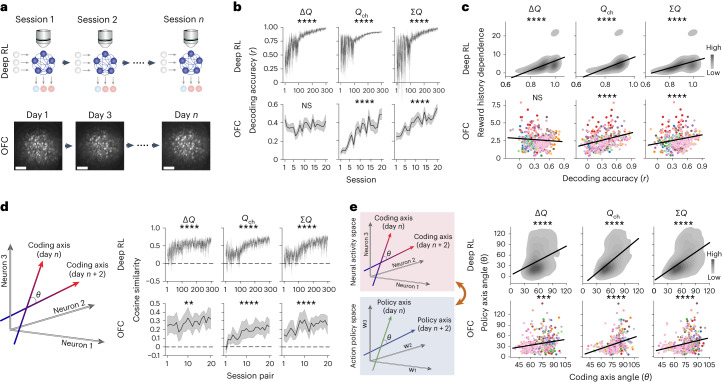
Dynamics and stabilization of OFC value coding during meta-learning. **a**, Longitudinal tracking of neural populations across sessions (5 deep RL models, 14 OFC populations). Scale bar = 100 µm. This figure focuses on the activity at postchoice period (0–1 s for mice, the time point after choice for deep RL). Analyses at other trial periods are shown in Extended Data Figs. 9 and 10. **b**, Decoding accuracy of value-related signals increases during training. All 100 recurrent units were used for deep RL models, and OFC neurons were subsampled (55 cells per population). Shadings indicate s.e.m. Statistics are from mixed effects models with session as the fixed effect and neural population as the random intercept. Deep RL (Δ*Q*, *P* = 7.08 × 10^−202^; *Q*_ch_, *P* = 1.72 × 10^−99^; ∑*Q*, *P* = 1.21 × 10^−110^), mouse (Δ*Q*, *P* = 0.22; *Q*_ch_, *P* = 8.53 × 10^−19^; ∑*Q*, *P* = 3.24 × 10^−14^). **c**, Relationships between the decoding accuracy and the strength of behavioral dependence on reward history (sum of unsigned regression weights). Kernel density estimation of the distributions (deep RL), and scatterplots with different colors for 14 different OFC populations. For deep RL, early sessions (<100th) were excluded due to their unstable decoding accuracy. Regression lines and statistics are from mixed effects models (accuracy as the fixed effect, neural population as the random intercept). Deep RL (Δ*Q*, *P* = 3.92 × 10^−73^; *Q*_ch_, *P* = 1.76 × 10^−63^; ∑*Q*, *P* = 6.72 × 10^−58^), mouse (Δ*Q*, *P* = 0.13, *Q*_ch_, *P* = 6.75 × 10^−16^; ∑*Q*, *P* = 1.26 ×;10^−6^). **d**, Angle between coding axes for shared neurons from adjacent sessions (1 session apart for deep RL, 2 d apart for OFC) was measured to quantify the similarity of population coding for value-related signals. Cosine similarity of the coding axes increases during training in both deep RL and mouse OFC. Shadings indicate s.e.m. Statistics are from mixed effects models with session pair as the fixed effect and neural population as the random intercept. Deep RL (Δ*Q*, *P* = 2.18 × 10^−39^; *Q*_ch_, *P* = 6.14 × 10^−140^; ∑*Q*, *P* = 1.53 × 10^−154^), mouse (Δ*Q*, *P* = 2.55 × 10^−3^; *Q*_ch_, *P* = 7.53 × 10^−11^; ∑*Q*, *P* = 3.13 × 10^−7^). **e**, Relationships between the angle of coding axes for values and the angle of action policy axes for reward history in pairs of sessions. The similarity in coding axes correlates with the similarity in behavioral action policies. Deep RL (Δ*Q*, *P* = 3.60 × 10^−39^; *Q*_ch_, *P* = 4.35 × 10^−113^; ∑*Q*, *P* = 3.78 × 10^−102^), mouse (Δ*Q*, *P* = 9.83 × 10^−4^; *Q*_ch_, *P* = 3.56 × 10^−10^; ∑*Q*, *P* = 3.02 × 10^−6^). Statistics are from mixed effects models with coding axis angle as the fixed effect and neural population as the random intercept. NS *P* > 0.05, **P < 0.01, ***P < 0.001, ****P < 0.0001. All tests are two-sided. Source data

**Extended Data Fig. 9:**
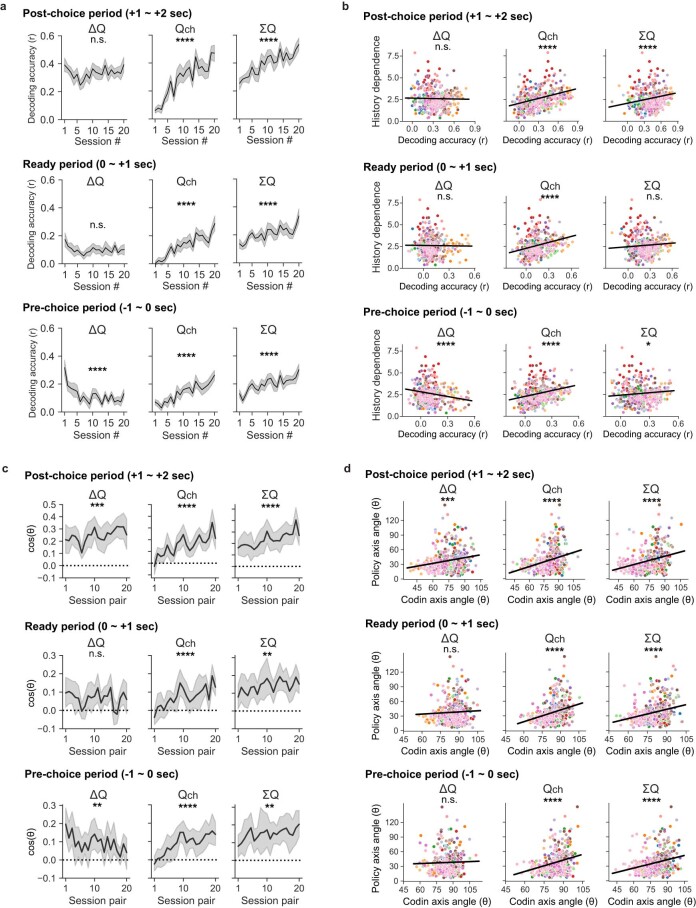
Relationships between behavioral action policy and value coding in OFC neural activity at different trial periods. **a**, Decoding accuracy of value-related signals across training days. Coding of Q_ch_ and ∑Q consistently increased during training across all trial periods. ΔQ signal during later trial periods decreased in early training sessions, possibly reflecting that OFC is not the primary site for within-trial maintenance of this information. Areas such as the retrosplenial cortex may be responsible for the within-trial maintenance^7,8^. OFC neurons were subsampled (55 cells/population) for decoding. Statistics are from mixed effects models with session as the fixed effect and neural population as the random intercept. Post-choice (ΔQ: p = 0.35, Q_ch_: p = 2.07 × 10^−20^, ∑Q: p = 3.05 × 10^−12^), ready (ΔQ: p = 0.33, Q_ch_: p = 4.05 × 10^−18^, ∑Q: p = 5.65 × 10^−7^), pre-choice (ΔQ: p = 5.81 × 10^−7^, Q_ch_: p = 4.63 × 10^−14^, ∑Q: p = 3.84 × 10^−8^). **b**, Relationships between the decoding accuracy and the strength of behavioral dependence on reward history (Sum of unsigned regression weights). Scatterplots with different colors for 14 different OFC populations. Statistics are from mixed effects models with accuracy as the fixed effect and neural population as the random intercept. Post-choice (ΔQ: p = 0.68, Q_ch_: p = 2.07 × 10^−17^, ∑Q: p = 3.23 × 10^−6^), ready (ΔQ: p = 0.80, Q_ch_: p = 2.33 × 10^−11^, ∑Q: p = 0.081), pre-choice (ΔQ: p = 3.13 × 10^−5^, Q_ch_: p = 6.93 × 10^−10^, ∑Q: p = 0.019). **c**, Angle between coding axes for shared neurons from adjacent sessions (2 days apart for OFC) was measured to quantify the similarity of population coding for value-related signals. Cosine similarity of the coding axes increases during training except for the ΔQ at ready and pre-choice periods (likely due to weak ΔQ signal during these trial periods as shown in (**a**)). Statistics are from mixed effects models with session pair as the fixed effect and neural population as the random intercept. Post-choice (ΔQ: p = 4.35 × 10^−4^, Q_ch_: p = 1.09 × 10^−12^, ∑Q: p = 3.99 × 10^−7^), ready (ΔQ: p = 0.37, Q_ch_: p = 1.20 × 10^−7^, ∑Q: p = 5.94 × 10^−3^), pre-choice (ΔQ: p = 1.89 × 10^−3^, Q_ch_: p = 8.63 × 10^−9^, ∑Q: p = 7.92 × 10^−3^). **d**, Relationships between the angle of coding axes for values and the angle of action policy axes for reward history in pairs of sessions. The similarity in coding axes correlates with the similarity in behavioral action policy except for ΔQ at ready and pre-choice periods (likely due to weak ΔQ signal at these trial periods). Statistics are from mixed effects models with coding axis angle as the fixed effect and neural population as the random intercept. Post-choice (ΔQ: p = 7.31 × 10^−4^, Q_ch_: p = 1.34 × 10^−8^, ∑Q: p = 3.15 × 10^−7^), ready (ΔQ: p = 0.36, Q_ch_: p = 8.14 × 10^−8^, ∑Q: p = 1.57 × 10^−5^), pre-choice (ΔQ: p = 0.54, Q_ch_: p = 5.39 × 10^−8^, ∑Q: p = 2.77 × 10^−5^). All shadings indicate s.e.m. All regression lines and statistics are from mixed effects models (Methods). n.s. P > 0.05, **P < 0.01, ***P < 0.001, ****P < 0.0001. All tests are two-sided. Source data

The results so far indicate that CaMKII-dependent plasticity in OFC is required for efficient across-session meta-learning of RL action policy but not for the trial-by-trial RL performance by expert mice, while OFC activity is required for expert trial-by-trial RL performance. These results resemble the deep RL model in which the plasticity-based outer-loop RL slowly establishes the inner-loop algorithm that performs trial-by-trial RL using recurrent activity dynamics (Fig. 1g). To investigate how the inner-loop activity-based RL is modified by the outer-loop plasticity-based RL during training, we longitudinally tracked identical neural populations across training sessions in both deep RL models and mice (Fig. 7a). We found that the strength of the three value-related signals increased in the recurrent activity of deep RL models during training (Fig. 7b), and the behavioral dependence on reward history closely tracked the strength of the value-related signals (Fig. 7c). OFC similarly increased *Q*_ch_ and ∑*Q* signals in the population activity during training (Fig. 7b and Extended Data Fig. 9a), and the behavioral history dependence tracked the signal strength (Fig. 7c and Extended Data Fig. 9b). Δ*Q* signal strength in OFC did not correlate with the behavioral history dependence in mice unlike in deep RL models, suggesting that Δ*Q* signal strength in OFC is not the limiting factor to determine behavioral dependence on this signal.

To evaluate the evolution of action value representations during across-session meta-learning, we longitudinally examined the coding axes for value-related signals in neural population activity. For each mouse and deep RL model, we identified the coding axes for the three value-related signals and defined the similarity of the coding axes between nearby sessions as the cosine similarity between the two axes. In both deep RL model and mouse OFC, we found that the stability of coding axis was initially low but gradually increased across training sessions (Fig. 7d and Extended Data Fig. 9c), indicating a highly dynamic reorganization at the early phase of training and a gradual stabilization of value coding over training sessions.

**Extended Data Fig. 10:**
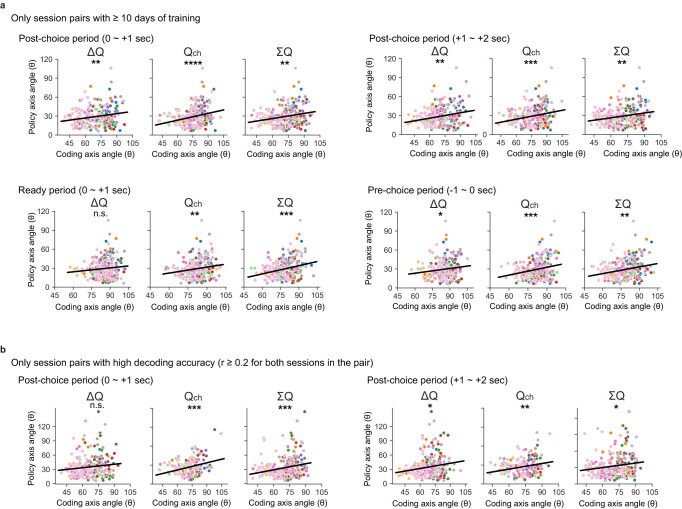
Relationships between value coding axis and action policy axis are not due to the noisy estimates of axes in early training sessions. **a**, Relationships between the similarity of value coding axes and the similarity of action policy axes for reward history are shown only for the session pairs with at least 10 days of training. The relationships remain after excluding early sessions with poor behavioral performance. Post-choice 0 ~ +1 sec (ΔQ: p = 9.92 × 10^−3^, Q_ch_: p = 8.78 × 10^−5^, ∑Q: p = 2.21 × 10^−3^), Post-choice +1 ~ +2 sec (ΔQ: p = 2.09 × 10^−3^, Q_ch_: p = 3.98 × 10^−4^, ∑Q: p = 4.25 × 10^−3^), ready (ΔQ: p = 0.12, Q_ch_: p = 7.70 × 10^−3^, ∑Q: p = 2.03 × 10^−4^), pre-choice (ΔQ: p = 4.46 × 10^−2^, Q_ch_: p = 2.71 × 10^−4^, ∑Q: p = 1.25 × 10^−3^). **b**, Relationships between the similarity of value coding axes and the similarity of action policy axes for reward history are shown only for the session pairs with at least r = 0.2 decoding accuracy for both sessions in the pair. The relationships remain after excluding the pairs with noisy value coding axes. Post-choice 0 ~ +1 sec (ΔQ: p = 0.10, Q_ch_: p = 1.27 × 10^−4^, ∑Q: p = 9.77 × 10^−4^), Post-choice +1 ~ +2 sec (ΔQ: p = 0.018, Q_ch_: p = 4.15 × 10^−3^, ∑Q: p = 0.020). All regression lines and statistics are from mixed effects models (coding axis angle as the fixed effect and neural population as the random intercept, two-sided). n.s.: P > 0.05, *P < 0.05, **P < 0.01, ***P < 0.001, ****P < 0.0001. Source data

The stabilization of value coding may lead to a stabilization of the action policy to compute value from history information, thus resulting in a more stable use of history to drive decision-making. A prediction of this idea is that behavioral action policies also stabilized during training, tracking the stabilization of value coding. We tested this prediction by examining whether the similarity of value coding axes between pairs of sessions correlates with the similarity of behavioral action policies for reward history. In both deep RL model and OFC, the coding axis similarity of paired sessions positively correlated with the policy axis similarity (Fig. 7e and Extended Data Fig. 9d). This relationship remained even when we used only later sessions (≥10 d of training) or only sessions with high decoding accuracy (*r* ≥ 0.2; Extended Data Fig. 10).

The close relationship between OFC value coding and behavioral action policies supports a critical role of OFC in guiding meta-RL. Taken together, these results indicate that the meta-RL mechanism in the mouse brain resembles the deep RL model with a meta-RL framework, with the OFC having a central role in both fast and slow timescales of RLs.

## Discussion

Recurrent network activity and long-term synaptic plasticity are two distinct mechanisms by which neural networks can process and store information. Because of the difference in the underlying mechanisms, they can run in parallel in a shared neural network at distinct timescales. Deep RL models with meta-RL frameworks take advantage of this flexibility and implement slow and fast RL^3–6,50^. Here we showed that CaMKII-dependent plasticity in OFC is required for slow but not fast RL, while OFC activity is required for fast RL, resembling the deep RL models. Put it another way, OFC uses two distinct mechanisms at two timescales to mediate slow and fast RL. The multiple layers of learning with distinct mechanisms and timescales would confer an extra level of flexibility and stability in cognition and learning^2,6^. The slow plasticity-based learning allows the accumulation of experiences over long periods of time, leading to the storage of generalized knowledge as synaptic weights. Animals can then exploit this stable and generalized knowledge to quickly adapt to new environments, using computations by the recurrent networks.

Although our work demonstrates the critical involvement of OFC in both fast and slow RL, many other brain regions are also involved in RL^7,8,10,16,29,51–55^. OFC likely works together with these other regions that mediate different aspects of fast trial-by-trial RL, such as stable maintenance^7,8,53^ and updating^29,54,55^ of values. Our results indicate that OFC has at least the functions of the actor network in the deep RL model, but the functions of the critic network (evaluation of the ongoing action policy) may be mediated by other areas such as other prefrontal areas, striatum and ventral tegmental area. Furthermore, plasticity-based RL may happen at different speeds in different brain areas, possibly governed by differences of neuromodulatory inputs and their receptor expression^5,23,25,50^. Even a single area may possibly run multiple timescales of plasticity-based RL in parallel using distinct mechanisms (for example, long-term plasticity versus short-term plasticity). Different timescales of plasticity within and across areas may confer extra flexibility on the brain by providing more than two timescales for RL. Future studies will unravel how different regions work together to regulate the meta-RL process in the brain, which would inspire further modifications of meta-RL frameworks for deep RL models in the field of AI. Our study provides an important clue toward the neurobiological understanding of ‘learning to reinforcement learn’.

## Methods

### Animals

All procedures were in accordance with the Institutional Animal Care and Use Committee at University of California San Diego. Wild-type (WT) C57BL/6 mice were obtained from Charles River. Transgenic mice were obtained from the Jackson Laboratory (CaMKIIa-tTA: B6;CBA-Tg(Camk2a-tTA)1Mmay/J (JAX 003010); tetO-GCaMP6s: B6;DBA-Tg(tetO-GCaMP6s)2Niell/J (JAX 024742); PV-Cre: B6;129P2-Pvalb^tm1(cre)Arbr^/J (JAX 008069)). All surgeries and in vivo experiments were carried out in adult mice (6 weeks or older). Mice were housed in a cage (68–72 °F temperature and 0–100% humidity) and had free access to food. Mouse cages were kept in a room with a reversed 12-h light/12-h dark cycle, and all experiments were performed during the dark period. All postsurgery mice were singly housed with a running wheel. Both male and female healthy adult mice were used for most experiments except for the paAIP2 experiments where only male C57BL/6 adult mice were used.

### Surgery for two-photon imaging and optogenetics

Mice were injected with dexamethasone (2 mg kg^−1^) subcutaneously at the beginning of surgery and continuously anesthetized with 1–2% isoflurane during surgery. All surgeries were performed while mice were placed on heating pads, and their eyes were protected with Vaseline. After cleaning the surface of dorsal skull with a razor blade and 75% ethanol, we performed craniotomy at the target lateral OFC coordinate (~1.45 mm lateral and ~2.6 mm anterior from bregma) where we implanted either a GRIN lens or a fiber-optic cannula. The details of the procedures up to the craniotomy step can be found in ref. ^56^.

For two-photon imaging experiments, we first aspirated the cortex above the target coordinate up to 1.0 mm depth using a blunt end 30G needle (0.312 mm O.D.; SAI Infusion Technologies). Then, we unilaterally implanted a GRIN lens (Inscopix, GLP-0561; 500 µm diameter) above the deep layer of lateral OFC (1.5 mm depth) in either left or right hemisphere. Note that the layers are inverted in the ventral cortex, so we targeted the deep layer to keep all layers of OFC intact. The implanted GRIN lens was fixed at the target coordinates using 3M Vetbond (WPI)^57^ on the skull, followed by cyanoacrylate glue and black dental acrylic cement (Lang Dental). We glued the upper part of 1.5 mm screw cap tube (Thermo Fisher Scientific) along with its screw cap on the head using cyanoacrylate glue and black cement to protect the implanted GRIN lens.

For optogenetics experiments, we first bilaterally injected respective viruses in the OFC of both hemispheres (2.0 mm depth). For inactivation experiments, we injected ~200 nl of AAV5-Syn-FLEX-rc(ChrimsonR-tdTomato; Addgene) or AAV2/1-CAG-FLEX-tdTomato-WPRE (Addgene) in PV-Cre transgenic mice. For plasticity-blocking experiments, we injected ~350 nl of AAVDJ-CaMKIIP-mEGFP-P2A-paAIP2 (plasmid from Addgene, virus production by J.H.C. and B.K.L.) or AAV2/1-CB7-EGFP (Addgene) in WT C57BL/6 mice. After virus injections, we bilaterally implanted fiber-optic cannulas (0.22 NA, 200 µm fiber diameter, 1.5 mm fiber length for inactivation experiments—Newdoon and 0.66 NA, 400 µm fiber diameter, 1.5 mm fiber length for plasticity-blocking experiments–Doric Lenses) at 1.45 mm depth. Implanted fiber-optic cannulas were fixed at the target coordinates using 3M Vetbond (WPI)^57^ on the skull, followed by cyanoacrylate glue and black dental acrylic cement (Lang Dental).

Additionally, a custom-built metal head bar was secured on the skull above the cerebellum with cyanoacrylate glue and dental acrylic cement. Buprenorphine (0.1 mg kg^−1^ of body weight) and Baytril (10 mg kg^−1^ of body weight) were subcutaneously injected after surgery, and mice were monitored until they recovered from anesthesia.

### Surgery for extracellular spike recording

For extracellular spike recording experiments, we covered the dorsal skull with cyanoacrylate glue before craniotomy so that dental cement attaches well on the skull at a later step. Then, we made a small hole in the skull above cerebellum and implanted a ground wire (stainless steel). The implanted ground wire and a head bar were fixed on the cerebellum skull using cyanoacrylate glue and black dental acrylic cement (Lang Dental). Next, we performed another craniotomy above lateral OFC (lOFC: ~1.45 mm lateral and ~2.6 mm anterior from bregma) and unilaterally injected either ~350 nl of AAVDJ-CaMKIIP-mEGFP-P2A-paAIP2 or AAV2/1-CB7-EGFP in the right hemisphere of WT C57BL/6 mice. After virus injections, we made a small hole in the dura and slowly inserted a chronic 64-channel silicone probe (ASSY-236 H6 probe and mini-amp-64 from Cambridge NeuroTech: 2 shanks with 32 channels per shank, recording sites are tiled along 400 µm of each shank) along with a lambda optic fiber (0.39 NA, 200 µm fiber diameter, 700 µm active length; Optogenix) up to 1400 µm depth (the probe was slowly moved further down to the OFC during pretraining tasks). The distance between the recording sites and the active part of the fiber is ~220 µm. Both the probe and the fiber were mounted on a Nano-Drive V2 (Cambridge NeuroTech) before the implantation so that we could change the probe depth during training. After the implantation, the dura was sealed by Dura-Gel (Cambridge NeuroTech). Then, we covered the electrode and the sliding part of the Nano-Drive with Vaseline using low-temperature cautery (FIAB, F7255). After mounting sufficient amount of Vaseline, the implants were fixed on the skull using black dental acrylic cement (Lang Dental). Buprenorphine (0.1 mg kg^−1^ of body weight) and Baytril (10 mg kg^−1^ of body weight) were subcutaneously injected after surgery, and mice were monitored until they recovered from anesthesia.

### RL task and training for mice

Mice were water-restricted at 1–2 ml d^−1^. After at least a week of water restriction, we started behavioral training. Behavioral control was automated with a real-time system. We used BControl system (C Brody, Princeton University) running on Linux communicating with MATLAB (MathWorks) for imaging experiments, and Bpod system (v0.5, J. I. Sanders and A. Kepecs, Washington University in St. Louis) running on Arduino DUE communicating with MATLAB for optogenetics experiments. For the Bpod system, another dedicated Arduino UNO with a sound card (Adafruit, ADA1788) was also used to generate sounds from a speaker. We wrote custom behavior scripts on respective systems for our behavior task. We previously reported this behavior task and training^7^. Mice were head-fixed on a custom-built behavior stage. Two lickports were placed on the left and right sides of the head-fixed mouse, and licking was monitored by infrared radiation (IR) beams. An amber LED for the ready cue was placed ~5 cm away from the nose, and a speaker was placed under the mouse stage. Each trial has a ready period, an answer period and ITI. At the beginning of each trial, the amber LED turned on to signal the beginning of the ready period. The ready period lasted for either 2 or 2.5 s (randomly assigned every trial). After the ready period, the speaker generated 10 kHz tone for the go cue. Both the 10 kHz tone and amber LED cues were terminated when mice made a choice (the first lick to one of the lickports during the answer period) or when mice did not lick for the maximum answer period of 2 s. Each choice was accompanied by a 50 ms feedback tone (left, 5 kHz; right, 15 kHz). If a reward was assigned to the chosen side of the lickport, ~2 µl of water was released immediately after the choice. The answer period and choice were followed by ITI.

Before training mice in the probabilistic reversal learning task, we trained mice in three different pretraining tasks (pretask I: 2–3 d, pretask II: 2–5 d and pretask III: ~2 weeks). In the pretask I, all the choices mice made during the answer period were rewarded with 100% probability. Licking during the ready period was not punished in this pretraining phase, and the mean ITI was gradually increased from 1 to 6 s (±1 s jitter in the duration of every trial). Mice learn that they can collect rewards by licking lickports during the answer period in the pretask I. In the pretask II, reward was delivered alternately from left and right lickports following either choice during the answer period. From this pretask II, licking during the ready period was punished by 500 ms white noise alarm sound and trial abort with an extra 2 s ITI. Mice learn that they can collect rewards from either lickport and need to withhold licking during the ready period in the pretask II. In the pretask III, a choice was rewarded only when the choice was opposite to the choice in the immediately preceding trial. Mice were encouraged to choose from both lickports in this pretask III. Training in the pretask III was terminated when their correct choice rates reached 70%. Through these three pretraining tasks, mice learned the general task structure, including that only their first lick during the answer period is associated with outcome, rewards are available from both lickports and, they need to withhold licking during the ready period.

After the pretraining, we trained mice in the probabilistic reversal learning task. We assigned reward to each lickport on every choice trial according to a specific reward assignment probability of each lickport. In each trial, one of the lickports had a higher reward assignment probability. The combinations of reward assignment probabilities were either (60%, 10%) or (52.5%, 17.5%), and the probability changed randomly every 60–80 trials in the order of (left, right) = …, (60%, 10%), (10%, 60%), (52.5%, 17.5%), (17.5%, 52.5%), (60%, 10%), …. We postponed the probability block transition if the fraction of choosing the lickport with a higher reward assignment probability was below 50% in recent 60 trials until the fraction reached at least 50% to ensure that mice switch choice preference in each probability block. Once a reward was assigned to a lickport on a trial, the reward remained assigned to the lickport until the lickport was chosen in the future trial (concurrent variable-interval schedules^9,11,58^). ITI varied randomly between 5 and 7 s. Both alarm (trials with licking during the ready period) and miss (trials without licking during the answer period) trials were not rewarded. We did not include alarm and miss trials in neural activity analyses to ensure that the ready periods we analyzed were free of licking behaviors and that mice were engaged in the task in the trials.

### Artificial meta-RL network

We trained artificial meta-RL networks (deep RL models) with firing rate units in this study (five independently trained deep RL models). Learning rate of 0.0005 was used for the network training. The network architecture and training method of our artificial neural networks are based on previous papers on meta-RL^3–5^. Unlike the previous publications, we used simple recurrent units with tanh activation functions instead of gated recurrent units (LSTM cell^59^) because biological neurons do not have such sophisticated gated functions. Therefore, maintenance and computation of signals were performed only through recurrent connectivity. We trained the meta-RL network using the A2C method^12^ with a single worker. The number of time steps per trial was randomly assigned to 4 or 5 on each trial to reflect the variable ITI in the mouse task. The meta-RL network had three input neurons that each received the information of either reward outcome (1 for reward, 0 otherwise), left action (1 for left, 0 otherwise) or right action (1 for right, 0 otherwise) from the immediately preceding time step. These input neurons are inactive except for the single time step immediately after previous choice in each trial. These input neurons are connected to the next recurrent layer (50 units for actor and 50 units for critic), and the history signals are maintained through the recurrent connectivity in the recurrent layer. The output of either actor or critic is given by

1 $${{{\mathbf{y}}}}_{({{t}})}=\tanh ({{{W}}}_{{{\mathbf{x}}}}{{{\mathbf{x}}}}_{\left({{t}}\right)}+{{{W}}}_{{{\mathbf{y}}}}{{{\mathbf{y}}}}_{\left({{t}}-{\bf{1}}\right)}+{{\mathbf{b}}})$$

where $\tanh (\bullet )$ is a hyperbolic tangent activation function of the form $\tanh \left(z\right)=\frac{{e}^{z}-{e}^{-z}}{{e}^{z}+{e}^{-z}}$, **x**_(t)_ is a 3 × 1 vector representing the inputs from the presynaptic three input neurons at a time step *t*, **y**_(*t*−1)_ is a 50 × 1 vector representing the layer’s outputs from a previous time step (*t* − 1), *W*_**x**_ is a 50 × 3 matrix containing the connection weights for the inputs from three presynaptic input neurons, *W*_**y**_ is a 50 × 50 matrix containing the connection weights for the recurrent connections and **b** is a 50 × 1 vector containing each neuron’s bias term. The recurrent neurons in the actor network project to two output neurons, and the recurrent neurons in the critic network project to one output neuron. The two output neurons from the actor represent logits for left and right actions, and the actions were sampled from the softmax distribution defined by these action logits outputs. Because the input neurons send history signals to the recurrent layer only at the first time step immediately after previous choice, the action selection of the network needs to rely on the history signals that were maintained in the recurrent layer across time steps through its recurrent connectivity. The selected action accompanies a reward if the reward was loaded on the selected side in the trial. On the other hand, an output neuron from the critic represents the state value for the next trial. Following the A2C method, we defined the policy loss (${L}_{\pi }$) and value loss (${L}_{v}$) as follows:

2 $${L}_{\pi }=-\mathrm{ln}\pi \left({a}_{t},|,{s}_{t}\right)\times A\left({s}_{t}\right)-{\beta }_{e}\times H\left(\pi \right)$$

3 $${L}_{v}={\beta }_{v}\times 0.5\times {\left({R}_{t}-V\left({s}_{t}\right)\right)}^{2}$$

where ${a}_{t}$ is the action, ${s}_{t}$ is the state, $\pi \left({a}_{t},|,{s}_{t}\right)$ is the action policy, $A\left({s}_{t}\right)$ is the advantage function, $H\left(\pi \right)$ is the entropy of the policy, ${R}_{t}$ is the discounted *n*-step bootstrapped return that represents the expected future rewards and $V\left({s}_{t}\right)$ is the state value. ${\beta }_{e}$ and ${\beta }_{v}$ are the hyperparameters that determine the relative contributions of the entropy term and the value loss to the total loss function. We used TD error $\delta \left({s}_{t}\right)$ as the estimator of the advantage $A\left({s}_{t}\right)$ function as follows:

4 $$A\left({s}_{t}\right)=\delta \left({s}_{t}\right)={R}_{t}-V\left({s}_{t}\right)$$

The *n*-step return ${R}_{t}$ is given by

5 $${R}_{t}={r}_{t}+\gamma {r}_{t+1}+{\gamma }^{2}{r}_{t+2}+\cdots +{\gamma }^{n-1}{r}_{t+n-1}+{\gamma }^{n}V\left({s}_{t+n}\right)$$

The entropy of policy $H\left(\pi \right)$ is given by

6 $$H\left(\pi \right)=-\sum _{a}\pi \left({a}_{t},|,{s}_{t}\right)\mathrm{ln}\pi \left({a}_{t},|,{s}_{t}\right)$$

The parameters of the networks were updated following the gradients of the total loss function (${L}_{{\mathrm {tot}}}={L}_{\pi }+{L}_{v}$) as follows:

7 $$\begin{array}{c}\nabla {L}_{{\mathrm {tot}}}=-\frac{\partial \mathrm{ln}\pi \left(a,|,s\right)}{\partial {\theta }_{a}}* A\left(s\right)-{\beta }_{e}\frac{\partial H\left(\pi \right)}{\partial {\theta }_{a}}+{\beta }_{v}* 0.5* \frac{\partial {\left(R-V\left(s\right)\right)}^{2}}{\partial {\theta }_{c}}\\ =-\frac{\partial \mathrm{ln}\pi \left(a,|,s\right)}{\partial {\theta }_{a}}* \delta \left({s}_{t}\right)-{\beta }_{e}\frac{\partial H\left(\pi \right)}{\partial {\theta }_{a}}-{\beta }_{v}\delta \left({s}_{t}\right)\frac{\partial V\left(s\right)}{\partial {\theta }_{c}}\end{array}$$

where ${\theta }_{a}$ and ${\theta }_{c}$ represent the parameters for the actor and the critic networks, respectively. The networks were trained using RMSProp and backpropagation through time. The training hyperparameters were fixed as follows: learning rate = 0.0005, $\gamma$ = 0.5, ${\beta }_{e}$ = 0.5, ${\beta }_{v}$ = 0.01 and unroll length = 50 time steps. The networks performed 500 trials per session (episode) in the task environment where the reward assignment probabilities and its baiting rule are identical to the mouse task, and the network parameters were fixed within each session (no synaptic plasticity). Network parameters were updated after each training session to approximate slow learning. If weights are updated every trial, the networks learn to perform trial-by-trial RL using the weight updating mechanism as in standard deep RL models (for example, Deep Q-Network (DQN))^12,60^. Instead, we updated the weights of the networks infrequently to encourage the networks to learn activity dynamics-based trial-by-trial RL. Each network ran 300 training sessions and an additional 301st session of the network from the last training session.

For inactivation of a trained network, we randomly sampled specified fractions of neurons (0–100%) from the recurrent layer of the trained network and inactivated those neurons at the time step immediately before action selection to mimic the prechoice period inactivation experiments for mice. For each inactivation fraction condition, we repeated the simulations 50 times with different random subsampling of recurrent neurons for inactivation and averaged the results. Following the inactivation condition for OFC, we set the frequency of inactivation trials per session to ~13% with the constraint that each inactivation trial must be followed by at least four consecutive control trials.

### Two-photon calcium imaging of OFC neurons

In vivo neural calcium signals were recorded using two-photon microscopes (B-Scope; Thorlabs) with a ×16, 0.8 NA water immersion objective lens (Nikon) and 925 nm lasers (Ti-Sapphire laser; Newport). ScanImage (Vidrio Technologies) running on MATLAB (MathWorks) was used for image acquisitions. Images (512 × 512 pixels) were continuously recorded at ~30 Hz during task performance. We imaged from seven CaMKIIa-tTA::tetO-GCaMP6s transgenic mice expressing GCaMP6s in CaMKII-positive neurons. Signals were collected from deep layers of lateral OFC using unilaterally implanted GRIN lens (Inscopix, GLP-056; 1500 µm diameter). For each animal, we imaged two nonoverlapping neural populations from two different depths by alternately imaging at the two depths across training sessions. Therefore, we longitudinally imaged 14 distinct OFC neural populations (7 mice × 2 planes) in total. The positions of focal planes were adjusted before each imaging session such that the vasculature patterns and cells within the field-of view (FOV) were aligned to the template images from previous imaging sessions. Before starting each imaging session, we unscrewed the protective cap (Surgery for two-photon imaging and optogenetics) and attached a custom-built water chamber on the head to keep enough water between the GRIN lens and the objective lens during imaging. Slow drifts in the imaging field were manually corrected during imaging. Residual motions and image distortions were corrected by PatchWarp^61^. We used Suite2p^62^ to draw regions of interests (ROIs) corresponding to individual neurons and extract their fluorescence. The cellular ROIs were first classified by a user-trained classifier, and the classifications were further manually refined by humans. The pixels where multiple neurons overlap were excluded at the signal extraction step. Contaminations of neuropil activity in each cellular ROI were also estimated and removed from the extracted fluorescence following the algorithm on Suite2p. Slow linear trend was removed from each extracted fluorescence, and the detrended signal was deconvolved using a nonnegative deconvolution algorithm^41,42^ to obtain the estimate of the underlying spiking activity. To identify the same neurons between paired imaging sessions, we aligned their mean-intensity images along with cellular ROIs using affine transformations and manually identified ROIs corresponding to identical neurons between the paired sessions.

In this study, we analyzed the activity of a total of 47,311 OFC neurons (for this reported number, longitudinally imaged neurons were counted multiple times) from 390 imaging sessions in total. The average number of imaging sessions per population was 27.86 ± 7.73 (mean ± s.d.). The average number of analyzed cells per population for all imaging sessions was 171.76 ± 18.42, 127.67 ± 11.46, 116.5 ± 17.19, 89.17 ± 10.65, 172.06 ± 28.15, 99.60 ± 18.93, 172.57 ± 32.92, 128.84 ± 16.77, 151.41 ± 19.24, 104.70 ± 13.03, 114.62 ± 9.22, 111.58 ± 9.30, 96.73 ± 10.99 and 77.89 ± 10.83 for each of the 14 populations (mean ± s.d.).

### Optogenetic suppression of OFC activity

We performed OFC inactivation by optically activating PV-positive inhibitory neurons in OFC. We virally expressed ChrimsonR-tdTomato in Cre-dependent manner in PV-positive inhibitory neurons (Surgery for two-photon imaging and optogenetics). As the control mice, we virally expressed tdTomato without ChrimsonR in PV-positive inhibitory neurons. After head fixation, implanted fiber-optic cannulas were connected to 625 nm fiber-coupled LEDs (Thorlabs) using a bifurcated optic fiber. We also attached a red LED on the side of each of the fiber-optic cannulas to use it as the masking light by illuminating the mouse head every trial. We controlled the three LEDs (one for inactivation and two for masking light) to generate sequences of square pulses (40 Hz) using Arduino UNO. At the end of each stimulation period, we attenuated the intensity of all three LEDs with a linear attenuation over the last 100 ms. The intensity after the fiber-optic cannulas for OFC inactivation light was ~1.5 mW per fiber. We set the frequency of inactivation trials per session to ~13% with the constraint that each inactivation trial must be followed by at least four consecutive control trials to avoid excessive perturbation. In contrast to the LED for inactivation, the masking light LEDs were turned on every trial. The timing and duration of masking light LEDs on each trial were matched to those of the inactivation LED for each inactivation condition. Our inactivation covered both ITI and ready period (ITI + ready: from 0.5 s after the beginning of ITI until the end of the ready period), or briefly at either ITI (2 s ITI: from 1 s after the beginning of ITI until 3 s after the beginning of ITI, 5 s ITI: from the beginning of ITI until 5 s after the beginning of ITI) or ready period (ready: the entire ready period of 2 or 2.5 s). The numbers of mice and sessions collected for each condition were as follows: (ChrimsonR-tdTomato, ITI + ready, bilateral (6 mice, 43 sessions)), (ChrimsonR-tdTomato, 2 s ITI, bilateral (9 mice, 60 sessions)), (ChrimsonR-tdTomato, 5 s ITI, bilateral (8 mice, 47 sessions)), (ChrimsonR-tdTomato, ready, bilateral (10 mice, 62 sessions)), (tdTomato, ITI + ready, bilateral (5 mice, 30 sessions)), (tdTomato, 2 s ITI, bilateral (8 mice, 49 sessions)), (tdTomato, 5 s ITI, bilateral (8 mice, 46 sessions)), (tdTomato, ready, bilateral (8 mice, 49 sessions)) and (ChrimsonR-tdTomato, ITI + ready, unilateral (4 mice, 177 sessions)).

### Optogenetic suppression of OFC plasticity during behavior

We suppressed synaptic plasticity in OFC by optically activating paAIP2 (refs. ^31–33^), a photoactivatable inhibitor of CaMKII kinase activity, expressed in OFC neurons. We virally expressed EGFP-P2A-paAIP2 in CaMKII-positive neurons (Surgery for two-photon imaging and optogenetics). For control experiments, we virally expressed EGFP without paAIP2. After head fixation, implanted fiber-optic cannulas were connected to 473 nm blue lasers (Shanghai Laser & Optics Century Co.) via fiber-optic patch cords. We also attached a blue LED on the side of each of the fiber-optic cannulas to use it as the masking light by illuminating the mouse head every trial. Both the lasers and masking light LEDs generated continuous light. The laser intensity after the fiber-optic cannulas was ~25 mW per fiber. We bilaterally illuminated OFC in both EGFP-P2A-paAIP2 mice and EGFP control mice for the first 3 s during ITI on every trial. For the experiments where we blocked plasticity across training sessions, we split each WT male litter into half for paAIP2 (five mice) and control (five mice) group. The type of virus (EGFP-paAIP2 or EGFP) injected in each mouse was kept blind to the mouse trainer.

For the experiments where we started plasticity blocking at the expert stage, we trained a separate cohort of five paAIP2-expressing mice until they reached the expert stage using only masking blue LED light during the task performance. For each of the two types of history (reward and choice), we calculated the s.d. of the sum of history regression weights (five weights from Eq. (12)) during the recent 7 d of training sessions, and we judged the mouse as an expert with stable performance when the s.d. for all two types of summed history weights were <0.025 in the recent 7 d and the mouse had been trained for at least 17 d. We started the plasticity-blocking experiments after individual mice passed this criterion. During the experimental sessions, we bilaterally illuminated OFC through cannulas on alternating days (10 d of paAIP2 photoactivation sessions and 10 d of control sessions).

Although we blocked CaMKII activity only during behavior sessions, some task-related plasticity that normally occurs after a behavior session may be also suppressed. We previously found that synaptogenesis in M1 during motor learning, such as formation of new dendritic spines, tends to occur between days of learning, rather than during behavioral sessions^35^. The potent paAIP2 effects on M1 spine dynamics in the same lever-press task (Extended Data Fig. 3) suggest that the CaMKII blocking during behavioral sessions may also suppress task-related plasticity that occurs with some delays after each behavior session.

### Extracellular spike recording of OFC neurons

In vivo neural spikes were recorded using 64-channel chronic silicon probes (ASSY-236 H6, mini-amp-64; Cambridge NeuroTech) and a data acquisition board (Open Ephys) from a mouse with EGFP-P2A-paAIP2 expression and a mouse with only EGFP expression. Open Ephys GUI was used to monitor and save the recorded data at 30 kHz. The implanted probe and optic fiber were slowly moved down into OFC using Nano-Drive V2 (Cambridge NeuroTech) during the pretraining task. Blue laser light was delivered into OFC through the implanted fiber using the protocol we used for the behavior experiments (~25 mW, 3 s during ITI on every trial). After 30 d of illumination sessions, we recorded several distinct OFC populations by moving the probe depth. Our recorded neurons are from both deep and superficial layers (depth range: 1,500–2,100 µm). We used Kilosort3.0 (ref. ^63^) for automatic spike sorting, and the results were further manually refined using phy^64^.

### Organotypic OFC slice cultures

OFC slices were prepared from postnatal 4- to 6-d-old C57BL/6 mice, as described previously^65^. In brief, 350 μm-thick coronal cortical slices were prepared using a tissue chopper. Slices were placed on Millicell membranes (Millipore) in a culture medium containing minimal essential medium (Life Technologies), 20% horse serum, 1 mM l-glutamine, 1 mM CaCl_2_, 2 mM MgSO_4_, 12.9 mM d-glucose, 5.2 mM NaHCO_3_, 30 mM HEPES, 0.075% ascorbic acid and 1 μg ml^−1^ insulin, which was changed every other day. Slices were incubated at 37 °C in 5% CO_2_. Cortical slices were virally infected with 1 µl AAV mixture per slice (containing AAV9-Camk2a-Cre at 2 × 10^12^ vg ml^−1^ and AAV8-CBA-DIO-mEGFP-P2A-paAIP2 at 4.2 × 10^12^ vg ml^−1^) at DIV 4–6 and imaged or patched at DIV 10–13.

### Two-photon glutamate uncaging and light stimulation

Two-photon imaging was performed using a custom-built two-photon microscope. mEGFP was excited with a Ti:Sapphire laser (Coherent Ultra II) tuned at the wavelength of 920 nm. The fluorescence was collected by an objective lens (×60, 1.0 NA; Olympus) and detected with photoelectron multiplier tubes (Hamamatsu, H7422-40p) placed after wavelength filters (Chroma, HQ520/60 m-2p for green). The signal was acquired using a photon counting board (PicoQuant TH260) and custom software. A second Ti:Sapphire laser (InSight, Spectra-Physics) tuned at the wavelength of 720 nm was used to uncage 4-methoxy-7-nitroindolinyl-caged-l-glutamate (MNI-caged glutamate; Tocris) in artificial cerebral spinal fluid (ACSF) with a train of 6 ms, ~2.5–3 mW pulses (30 times at 0.5 Hz) near the spine of interest. In light stimulation experiments, slices were continuously illuminated with a blue LED (Thorlabs, M470L5) at a wavelength of 473 nm (160 mW cm^−2^) from the bottom of the sample. Experiments were performed at room temperature (∼25 °C), and slices were perfused with Mg^2+^ free ACSF (127 mM NaCl, 2.5 mM KCl, 4 mM CaCl_2_, 25 mM NaHCO_3_, 1.25 mM NaH_2_PO_4_ and 25 mM glucose) containing 1 μM tetrodotoxin and 4 mM MNI-caged l-glutamate aerated with 95% O_2_ and 5% CO_2_ at 25 °C.

### Patch-clamp electrophysiology

Infected OFC pyramidal neurons were visualized using epifluorescence illumination. Whole-cell current-clamp recordings were obtained using a Multiclamp 700B amplifier. Patch pipettes (3–5 ΩM) were filled with a potassium gluconate solution (130 mM K gluconate, 10 mM Na phosphocreatine, 4 mM MgCl_2_, 4 mM NaATP, 0.3 mM MgGTP, 3 mM l-ascorbic acid, 10 mM HEPES (pH 7.2) and 320 mOsm). These experiments were performed at room temperature (~25 °C) in ACSF containing 127 mM NaCl, 2.5 mM KCl, 2 mM CaCl_2_, 1 mM MgCl_2_, 25 mM NaHCO_3_, 1.25 mM NaH_2_PO_4_ and 25 mM glucose and oxygenated. Recordings were digitized at 10 kHz and filtered at 2 kHz. To test the effect of long-term blue light stimulation, whole-cell current-clamp recordings were performed in a different group of neurons before and after blue light stimulation for 40 min (1 s ON and 3 s OFF). Depolarizing current injections were given in 100 pA increments up to 800 pA. Threshold, action potential (AP) width and AP amplitude were analyzed on the current step where the first AP was observed. All data were acquired and analyzed with custom software written in C# and MATLAB.

### Two-photon imaging of spines over motor learning

WT (C57BL/6) mice between 3 and 6 months of age were prepared for craniotomy and cranial window placement, as previously described. Dendritic spines were visualized via sparse viral expression of either EGFP (AAV2/1.pCAG.FLEX.EGFP.WPRE.bGH; Allen Institute) or paAIP2-EGFP (AAV8-CBA-DIO-mEGFP-P2A-paAIP2) + diluted Cre recombinase (AAV9.pCaMKII.Cre, Penn Vector Core; 1:2,500–5,000× dilution in saline) in the forelimb region of the primary motor cortex, M1 (coordinates: +1,500 μm lateral, +300 μm anterior). Mice of different conditions were paired 1:1 with cage-matched siblings to minimize batch effects. Mice were allowed to recover with postoperative care for approximately 2 weeks, after which they were progressively water restricted—removing ad libitum access to water and reducing water delivered from 2 ml to 1 ml over 6 d—to achieve a maximum 30% reduction in body weight to motivate them to engage in the motor learning task. Water-restricted mice were acclimated to the behavioral imaging rig for 2 d before the experiment started and then subjected to the lever-press task. The apical dendrites of labeled layer 2/3 neurons were imaged via two-photon excitation of EGFP using a Ti:Sa pulsed laser tuned to 925 nm (MaiTai; Newport) coupled to a commercial two-photon microscope (B-Scope; Thorlabs) equipped with a ×16/0.8-NA objective (Nikon). Laser power was controlled with a Pockel’s cell and ranged from 7 to 40 mW. Imaging was always performed in awake animals. Images (512 × 512 pixels at zoom values ranging from 7 to 12.1×, corresponding to interpixel distances of 0.5 pixel per μm at 1× and scaling linearly for the zoom values used) were recorded at 30.05 Hz. Z-stacks of the apical dendritic arbor were taken by acquiring 100 frames per slice over a variable number of slices ranging from 20 to 60 with a step size of 1 μm, depending on the morphology and optical accessibility of targeted dendrites. This imaging process was repeated daily, before behavioral training, for each of the 14 sessions. Only data from the sessions indicated in Extended Data Fig. 3b are presented in this study.

### Lever-press task

Water-restricted mice were required to press a lever, comprising a piezoelectric flexible force transducer (LCL-113G; Omega Engineering) attached to a 1/14-mm-thick brass rod, past two thresholds (a ~1.5 mm lower threshold to prevent holding below baseline and a target threshold of ~3 mm) within 200 ms during the cue period (a 6 kHz tone) to receive a water reward (~10 μl). The lever position was continuously monitored using a data acquisition device (LabJack) and software (Ephus, MATLAB, MathWorks) working with custom software running on LabVIEW (National Instruments), which monitored threshold crossing. The behavioral setup was controlled with MATLAB software (Dispatcher, Z. Mainen and C. Brody). Rewarded trials were paired with a 500 ms, 10-kHz tone, while failed trials were presented with a white noise punishment signal and the start of the next intertrial interval (ITIs). ITIs were 8–12 s.

### Optical stimulation during motor learning

During training, light from a blue LED (465 nm; Doric) was directed through a 200 μm diameter patch chord into the cranial window by clamping from above (Extended Data Fig. 3a). Photostimulation occurred during the cue period of every trial in both paAIP2 and control mice. The average power measured at the fiber tip was ~2.5 ± 0.3 mW.

### Analysis of changes in dendritic spine size

Image stacks for each session were motion-corrected using a custom algorithm (MATLAB, MathWorks). This approach is similar to those reported previously^34–36^ but was co-opted to also register slices across *z* planes. This was achieved by first iteratively aligning all imaging frames within a given slice to the resulting frame average until performance saturated, then registering across slices from the central *z* slice moving to the top and bottom of the volume. To accurately assess spine volume without contamination from other structures (which is still possible despite sparse expression conditions), targeted dendrites were extracted via semi-automated tracing using the Simple Neurite Tracer plugin^66^ in ImageJ. This tool uses intensity-based path-finding to trace continuous paths along dendrites, after which approximately tubular volumes surrounding the dendrite can be considered, and intensity-based extractions of associated structures are achieved. Using this approach, large *z*-stacks of complicated dendritic arbor could be disentangled and analyzed individually. After extraction of target dendritic branches, segments of individual dendrites were selected for consideration based only on their consistent health and optical quality across sessions. It should be noted that optical obstructions could arise that did not reflect poor health; a portion of dendrite could be optically distorted on one imaging session, then appear in healthy and of high quality on a later session (Extended Data Fig. 3h, below red box). Dendritic segments that were selected for analysis based on these criteria were typically several 10 s of μm in length and displayed spine densities consistent with previous reports (Analysis of spine density and spine turnover). Maximum projection images of extracted dendrites were then used as the base images for subsequent measurements. Spine volume was measured by considering the integrated fluorescence intensity within an elliptical ROI drawn around the spine head in maximum projection images. To account for changing expression levels of the fluorescence indicator over days, integrated spine head intensities were normalized by the average dendritic intensity. Dendritic intensity was measured by drawing a series of points along the dendrite (which also allows the tracking of dendritic distance) and then expanding to an elliptical area around each point corresponding to the approximate diameter of the dendritic segment being analyzed. Unique individual pixels contained within these areas were then used as the ROI corresponding to a particular dendritic branch. To prevent over-generalization of dendritic normalization, only pixels within 20 μm (in dendritic distance) of a given spine were considered. These steps were repeated for both ‘early’ (session 1) and ‘late’ (sessions 11–14) imaging sessions. Late session images were selected based on optical quality of the imaged field, as described above. All analyses on a given imaging field used the same late session (that is, spines were counted only once and the late session was not decided on a ‘per-spine’ basis). The late sessions considered for EGFP-expressing (by mouse: 12, 13, 14, 14, mean = 13.25) and paAIP2-expressing (13, 14, 14, 11, mean = 13) animals were similar. Spine size estimates acquired in this way were largely stable over sessions (Extended Data Fig. 3e). The ‘enlargement’ threshold of 1.5× was chosen based on previous literature investigating controlled induction of spine enlargement in single spines.

### Analysis of spine density and spine turnover

In a separate set of analyses, spine density was measured along individual dendritic segments that were comparable in quality across sessions. These analyses used many of the same dendritic segments considered in the spine size measurements but were performed without specifically constraining the segments used in the size analysis. Spines were aligned based on the overall structural similarity of the surrounding region and were considered the same spine if (1) they presented similar structural appearance and (2) the attachment point of their neck to the dendrite occurred in a similar position relative to other spines and dendritic morphology. Spines were considered for analysis if and only if they could be easily discerned from other structures, which could lead to an underestimate of true spine density. Spine formation events were scored as those structures that did not have any structural correlates in session 1, while spine elimination events were those structures that were present in session 1 but had no obvious structural correlates in late sessions. ‘Stable’ spines correspond to all other spines; namely, those that were scored as ‘present’ throughout the experiment. While we acknowledge that very small-to-invisible spines may be inaccurately labeled as absent, such error is likely consistent across the groups being compared. Furthermore, our previous work tracking new spines in this way overlapped with orthogonally derived metrics that supported their identities as newly formed synapses^34^. To calculate spine densities, the linear distance along dendritic branches was extracted as the sum of linear distances between dendritic poly points (see description in Analysis of changes in dendritic spine size above). To measure the dendritic length in three-dimensional space, each dendritic poly point was assigned a slice address based on the maximum intensity of the extracted dendrite within the associated elliptical ROI. The distance between adjacent ROIs was thus calculated as either the linear distance between ROI centers or the hypotenuse between the linear distance and the *z*-step size (always 1 μm in the presented data), depending on whether they were addressed to the same *z* plane. Dendritic distances were measured between the locations of the ‘first’ and ‘last’ spines on a dendrite (that is, the flanking edges of spine counting for a given dendritic segment). We assumed that the dendritic length between registered spines is constant over sessions and used the dendritic length measured in session 1 for all sessions. These distances were used to calculate overall spine density, new spine density and elimination density.

### Assessment of dendritic health

Imaged dendrites were assessed for health based on standard parameters, such as spine density and dendritic morphology. ‘Blebbing,’ or large varicosities present along the dendrite accompanied by constriction of previously uniform regions, was considered reflective of a dead-or-dying cell and would instantiate the exclusion of such a dendrite from consideration in any session. Nonetheless, care was taken not to confuse blebs with spines oriented in the *z* axis, which can appear varicose along the dendrite. Such spines could be differentiated from blebs based on their size geometries in the *z* axis, as well as their commonplace appearance in early session images that were not yet exposed to risk of photodamage. These structures have previously been confirmed as spines with correlated electron microscopy of targeted dendrites^34^. Based on blebbing criteria alone, only one dendritic segment across the eight mice presented in this study was considered potentially sick, and this segment was excluded from all analyses. Given the potential effect of paAIP2 expression on spine density, we did not further filter dendrites based on spine density alone. The lack of significant difference between spine densities between EGFP- and paAIP2-expressing neurons (Extended Data Fig. 3i) is thus not a result of selection bias or unequal filtering of the data.

### Quantification of task performance in RL task

We used two types of performance metrics to quantify the task performance in each session. The first metric is the optimality score which quantifies how optimal the behavior was in each session. Once a reward was assigned to a lickport, the reward was maintained on the side until it was collected by a participant in our task environment. As a result, the probability that a reward is available on the lickport gradually increases if the lickport has not been selected in the recent trials^8^. Therefore, the probability that a reward is available on each side is given by

8 $${P}_{{\mathrm {rew}L}}(t)=1-\mathop{\prod }\limits_{x=t-{n}_{R}\left(t\right)}^{t}\left\{1-{A}_{L}(x)\right\}$$

9 $${P}_{{\mathrm{rew}R}}(t)=1-\mathop{\prod }\limits_{x=t-{n}_{L}\left(t\right)}^{t}\left\{1-{A}_{R}(x)\right\}$$

where ${A}_{c}(x)$ is the reward assignment probability of choice *c* on trial *x* (0.6, 0.525, 0.175 or 0.1), ${n}_{c}\left(t\right)$ is the number of successive *c* choices before trial *t* (for example, ${n}_{R}\left(t\right)=3$ when the choice on (*t* − 4) was left and the choices on (*t* − 3), (*t* − 2) and (*t* − 1) were right). Therefore, the optimal action policy that maximizes expected reward outcomes in our task environment is the policy that maximizes the following optimality score:

10 $${\mathrm {Optimality}}\,{\mathrm {score}}=\frac{1}{n}\mathop{\sum }\limits_{t=1}^{n}{P}_{{\mathrm {rew}C}}(t)$$

where $n$ is the number of trials and ${P}_{{\mathrm {rew}C}}(t)$ is the probability of reward availability on the chosen side in trial *t* (${P}_{{\mathrm {rew}L}}(t)$ in left choice trial and ${P}_{{\mathrm {rew}R}}(t)$ in right choice trial). The action policy with higher optimality score achieves higher reward rate in the task. Optimality score is a less noisy measure of behavioral optimality than a simple reward outcome rate score because the optimality score is not affected by the randomness of reward assignment in each trial.

The second performance metric is the frequency of choosing the side with higher reward assignment probability on each trial, which is given by

11 $$P\left({\mathrm {choosing}}\,{A}_{{\mathrm {High}}}\right)=\frac{\left({\rm{number}}\,{\mathrm {of}}\,{\mathrm {choices}}\,{\mathrm {with}}\,{A}_{{\mathrm {High}}}\left(t\right)\right)}{n}\,$$

where the numerator is the number of choices with higher reward assignment probability $A$ on each trial (${A}_{{\mathrm {High}}}$ is either 0.6 or 0.525). Although an action policy that maximizes this second metric is a suboptimal action policy in our task environment because of the cumulative nature of reward baiting, our mice primarily learned to increase this metric over the optimality score, which suggests that the objective function of the mouse learning may be closer to this second metric than the optimality score unlike deep RL models.

### Quantification of behavioral history dependence

We used a logistic regression model to quantify the behavioral history dependence of individual animals and deep RL models on each session. The model predicts an action on each trial based on reward and choice history from the past five trials^9^. The model is given by

12 $$\begin{array}{l}{\mathrm{ln}}\left(\frac{{P}_{L}\left(t\right)}{1-{P}_{L}\left(t\right)}\right)=\mathop{\sum }\limits_{i=1}^{5}{\beta }_{r\left(t-i\right)} \big({r}_{L}\left(t-i\right)-{r}_{R}\left(t-i\right)\big)\\+\mathop{\sum }\limits_{i=1}^{5}{\beta }_{c\left(t-i\right)} \big({c}_{L}\left(t-i\right)-{c}_{R}\left(t-i\right)\big)+{\beta }_{{\mathrm {bias}}}\end{array}$$

where ${P}_{L}\left(t\right)$ is the probability of choosing left on trial $t$, ${r}_{x}\left(t-i\right)$ is the reward history for left (*L*) or right (*R*) side on trial $t-i$ (1 for reward and 0 for no-reward), ${c}_{x}\left(t-i\right)$ is the choice history for left (*L*) or right (*R*) side on trial $t-i$ (1 for chosen and 0 for unchosen), ${\beta }_{r\left(t-i\right)}$ and ${\beta }_{c\left(t-i\right)}$ are the raw regression weights for each type of history and ${\beta }_{{\mathrm {bias}}}$ is the history-independent constant action bias term. We fit the model to behaviors using the L-BFGS solver without regularization (LogisticRegression function in scikit-learn^67^). Although we previously parametrized mouse behaviors in the same behavior task using three different types of history terms^7^, we now do not recommend the model because of the collinearity between the parameters.

### Action policy axis and magnitude of history dependence

To summarize the action policy in each behavior session, we defined action policy axes for the *k*th session as follows;

13 $$\vec{{{\boldsymbol{p}}}_{{\boldsymbol{r}}}^{{\boldsymbol{k}}}}=\left({\beta }_{r\left(t-1\right)}^{\,k},{\beta }_{r\left(t-2\right)}^{k},\ldots ,{\beta }_{r\left(t-5\right)}^{k}\right)$$

14 $$\vec{{{\boldsymbol{p}}}_{{\boldsymbol{c}}}^{{\boldsymbol{k}}}}=\left({\beta }_{c\left(t-1\right)}^{k},{\beta }_{c\left(t-2\right)}^{k},\ldots ,{\beta }_{c\left(t-5\right)}^{k}\right)$$

where $\vec{{{\boldsymbol{p}}}_{{\boldsymbol{r}}}^{{\boldsymbol{k}}}}$ and $\vec{{{\boldsymbol{p}}}_{{\boldsymbol{c}}}^{{\boldsymbol{k}}}}$ are the policy axes for each type of history. These axes are defined using the regression weights from Eq. (12). To quantify the stability of the action policy for each type of history across training sessions, we calculated the cosine similarity of the coding axis vectors for type-*x* history between *k*th session and (*k* + *m*)th session as follows:

15 $$\cos (\theta )=\frac{\overrightarrow{{{\boldsymbol{p}}}_{{\boldsymbol{x}}}^{{\boldsymbol{k}}}}\cdot \overrightarrow{{{\boldsymbol{p}}}_{{\boldsymbol{x}}}^{{\boldsymbol{k}}{\boldsymbol{+}}{\boldsymbol{m}}}}}{\Big\Vert\, \overrightarrow{{{\boldsymbol{p}}}_{{\boldsymbol{x}}}^{{\boldsymbol{k}}}}\Big\Vert \Big\Vert\, \overrightarrow{{{\boldsymbol{p}}}_{{\boldsymbol{x}}}^{{\boldsymbol{k}}+{\boldsymbol{m}}}}\Big\Vert }$$

where $\theta$ is the angle between the paired axis vectors in degrees and ||·|| denotes L2-norm of a vector.

### Quantification of inactivation effects on behavioral history dependence

To quantify the inactivation effects on the behavioral history dependence, we fit the following logistic regression model:

16 $$\begin{array}{c}\mathrm{ln}\left(\frac{{P}_{L}(t)}{1-{P}_{L}(t)}\right)\\ \,=\left(\mathop{\sum }\limits_{i=1}^{5}{\beta }_{r(i)}^{\mathrm{ctrl}} ({r}_{L}(t-i)-{r}_{R}(t-i)) \right.\\ \left.\qquad+\mathop{\sum }\limits_{i=1}^{5}{\beta }_{c(i)}^{\mathrm{ctrl}} ({c}_{L}(t-i)-{c}_{R}(t-i))+{\beta }_{\mathrm{bias}}^{\mathrm{ctrl}}\right)\times \mathrm{Ctrl}(t)\\ \,+\left(\mathop{\sum }\limits_{i=1}^{5}{\beta }_{r(i)}^{\mathrm{opto}} ({r}_{L}(t-i)-{r}_{R}(t-i))\right.\\ \left.+\mathop{\sum }\limits_{i=1}^{5}{\beta }_{c(i)}^{\mathrm{opto}} ({c}_{L}(t-i)-{c}_{R}(t-i))+{\beta }_{\mathrm{bias}}^{\mathrm{opto}}\right)\times \mathrm{Opto}(t)\end{array}$$

where ${P}_{L}\left(t\right)$ is the probability of choosing left on trial $t$, ${r}_{x}\left(t-i\right)$ is the reward history for left (*L*) or right (*R*) side on trial $t-i$ (1 for reward and 0 for no-reward), ${c}_{x}\left(t-i\right)$ is the choice history for left (*L*) or right (*R*) side on trial $t-i$ (1 for chosen and 0 for unchosen). ${\mathrm{Ctrl}}\left(t\right)$ is 1 on control trials and 0 on inactivation trials, while ${\mathrm{Opto}}\left(t\right)$ is 0 on control trials and 1 on inactivation trials. The model contains separate regression weights for control (${\beta }_{x}^{{\mathrm{ctrl}}}$) and inactivation (${\beta }_{x}^{{\mathrm{opto}}}$) trials. The model was fit to behaviors with L2 regularization (LogisticRegressionCV function in scikit-learn) to prevent overfitting to the data because the number of trials per fit was limited for these inactivation datasets. For the regularization, we selected the inverse of regularization strength from a logarithmic scale between 10^−4^ and 10^4^ (100 grids) by fivefold cross-validation. We used L-BFGS solvers for the L2 regularizations.

Because the frequency of inactivation trials is only ~13%, the number of control trials is much larger than the number of inactivation trials. To make the history dependence estimations robust against the difference in the trial numbers between control and inactivation trials, we matched the number of control trials to the number of inactivation trials for each model fitting by randomly subsampling the control trials. The subsampling and fitting were repeated with the smallest number of iterations to include every control trial at least once. We took the mean of the regression weights from all the iterations. For |Bias|, we took the absolute value of ${\beta }_{{\mathrm{bias}}}^{{\mathrm{ctrl}}}$ for each iteration before averaging across iterations.

### RL model

A class of RL models that originated from the Rescorla–Wagner (RW) model^13,68^ is widely used to estimate action values of animals and humans. Previously we optimized the RW RL model to describe the behavioral patterns of mice in the current task^7^. We used this model to estimate action values on each trial. In this RL model, the value of chosen action (${Q}_{{\mathrm {ch}}}$) is updated according to its reward outcome on every trial as follows:

17 $${Q}_{{\mathrm {ch}}}\left(t+1\right)=\left\{\begin{array}{c}{Q}_{{\mathrm {ch}}}\left(t\right)+{\alpha }_{{\mathrm {rew}}}\times \left(R\left(t\right)-{Q}_{{\mathrm {ch}}}\left(t\right)\right)\,\qquad\left({\mathrm {if}}\,{\mathrm {rewarded}},R\left(t\right)=1\right)\\ {Q}_{{\mathrm {ch}}}\left(t\right)+{\alpha }_{{\mathrm {unr}}}\times \left(R\left(t\right)-{Q}_{{\mathrm {ch}}}\left(t\right)\right)\qquad\left({\mathrm {if}}\,{\mathrm {unrewarded}},R\left(t\right)=0\right)\end{array}\right.$$

where ${R}\left(t\right)$ is reward outcome on trial $t$ (1 for rewarded and 0 for unrewarded trials), ${\alpha }_{{\mathrm {rew}}}$ is the learning rate for rewarded trials and ${\alpha }_{{\mathrm {unr}}}$ is the learning rate for unrewarded trials. Because the action value (*Q*) takes a value between 0 and 1, the reward prediction error $R\left(t\right)-{Q}_{{\mathrm {ch}}}\left(t\right)$ is positive on rewarded trials and negative on unrewarded trials.

The value of unchosen action (${Q}_{{\mathrm {unch}}}$) was also updated to reflect the time-dependent forgetting of unchosen action value^7,69,70^ as follows:

18 $${Q}_{{\mathrm {unch}}}\left(t+1\right)={\left(1-\omega \right)\times Q}_{{\mathrm {unch}}}\left(t\right)$$

where ${Q}_{{\mathrm {unch}}}$ is discounted every trial by the forgetting rate $\omega$.

We used the above value updating rule for both mice and deep RL models, but we used slightly different choice probability estimation to reflect the outcome-independent choice alternation that was unique in deep RL models (Fig. 1h). The probabilities of choosing left action (${P}_{L}$) for mice (Eq. 19) and deep RL models (Eq. 20) on trial $t$ are given by

19 $${P}_{L}\left(t\right)=\frac{1}{1+{e}^{-{\beta }_{\Delta Q}({{\beta }_{0}+Q}_{L}\left(t\right)-{Q}_{R}\left(t\right))}}$$

20 $${P}_{L}\left(t\right)=\frac{1}{1+{e}^{-{\beta }_{\Delta Q}({{\beta }_{0}+{\beta }_{c}C\left(t-1\right)+Q}_{L}\left(t\right)-{Q}_{R}\left(t\right))}}$$

where ${Q}_{L}$ and ${Q}_{R}$ are the action values for left and right, respectively, ${\beta }_{\Delta Q}$ reflects the sensitivity of a mouse to the action value difference, ${\beta }_{0}$ is the value-independent action bias that is constant in each session, $C\left(t-1\right)$ is the choice history on previous trial (1 for left choice and −1 for right choice) and ${\beta }_{c}$ is the weight for $C\left(t-1\right)$. Negative value on ${\beta }_{c}$ accounts for the tendency for choice alternation of deep RL models.

The cost function $J\left(\theta \right)$ was defined using the model likelihood $L\left(\theta \right)$ and L2-penalty as follows:

21 $$J\left(\theta \right)=-\mathrm{ln}L\left(\theta \right)+\frac{\lambda }{2}\mathop{\sum }\limits_{j=1}^{k}{\theta}_{\!j}^{2}$$

where ${\theta}_{\!j}$ represents the model parameters. L2-penalty was included to obtain a model with better generalization. The regularization parameter $\lambda$ was selected by tenfold cross-validation (minimum cross-validation error).

### Decoding of value-related signals

We decoded three different value-related signals, Δ*Q* (${Q}_{L}-{Q}_{R}$, value difference between left and right), ∑*Q* (${Q}_{L}+{Q}_{R}$, sum of the two action values) and *Q*_ch_ (value of the side chosen in the previous trial), from OFC neurons or the recurrent neurons in deep RL models. One important issue that needs to be considered for the decoding of value-related signals from neural activity is that action values are serially correlated across trials (autocorrelation). Because neural activity is also serially correlated, a simple decoder that decodes value on trial *t* from neural activity on trial *t* may overestimate the relationships between the value and neural activity. This is because two independent variables with slow serial correlations can appear correlated by chance^43–46^. Therefore, we devised a decoder that minimizes the contribution of the spurious correlations between slowly evolving variables. The spurious correlations originate from the autocorrelation of each variable across time. Therefore, we built the following decoders where majority of the slow autocorrelations of the value-related signals and the neural activity are ignored:

22 $${{\Delta}} Q\left(t+1\right)-{{\Delta}} Q\left(t\right)=\mathop{\sum }\limits_{i=1}^{n}{\beta }_{i}^{{{\Delta}} Q}\left({a}_{i}\left(t+1\right)-{a}_{i}\left(t\right)\right)+{\beta }_{0}^{{{\Delta}} Q}$$

23 $${Q}_{{\mathrm {ch}}}\left(t+1\right)-{Q}_{{\mathrm {ch}}}\left(t\right)=\mathop{\sum }\limits_{i=1}^{n}{\beta }_{i}^{{Q}_{{\mathrm {ch}}}}\left({a}_{i}\left(t+1\right)-{a}_{i}\left(t\right)\right)+{\beta }_{0}^{{Q}_{{\mathrm {ch}}}}$$

24 $$\varSigma Q\left(t+1\right)-\varSigma Q\left(t\right)=\mathop{\sum }\limits_{i=1}^{n}{\beta }_{i}^{\varSigma Q}\left({a}_{i}\left(t+1\right)-{a}_{i}\left(t\right)\right)+{\beta }_{0}^{\varSigma Q}$$

where ${a}_{i}\left(t\right)$ is the activity of the *i*th neuron on trial $t$, ${\beta }_{i}^{x}$ is the regression weight for the activity difference between adjacent choice trials and ${\beta }_{0}^{x}$ is the constant term. In each model, the difference in the value-related variable between adjacent choice trials is decoded using the neural activity difference between adjacent choice trials. This decoder focuses on the changes on each trial (trial derivatives). By focusing on the trial derivatives, we can suppress the potential spurious correlation between the value-related signal and neural population activity because trial derivatives have much less slow-timescale autocorrelations.

We used all recurrent neurons (*n* = 100) for decoding from deep RL models. The mean activity of the three time steps immediately before choice was used for the decoding from deep RL models. For decoding from OFC neurons (calcium imaging), we used only 55 neurons that were randomly subsampled from each population to match the number of neurons in the decoders across different sessions and mice. Fifty-five was the minimum number of simultaneously imaged neurons in our dataset. For each OFC neural population, we subsampled 55 neurons in each iteration without replacement until the iteration number reached the smallest number to include every cell for decoding. A small number of randomly selected neurons were sampled twice for the last iteration. The decoding accuracy from all iterations was averaged. Each iteration of decoding was performed using tenfold cross-validation without shuffling. For the decoding with intracellularly recorded spikes, we subsampled 18 neurons instead because the number of neurons that could be simultaneously recorded with a silicone probe was limited. We calculated chance decoding accuracy with two methods (within-session and cross-session). To obtain the within-session chance decoding accuracy, the value differences between adjacent trials were shuffled 100 times across trials. Decoding results from the shuffled data were averaged for the within-session chance decoding accuracy. The cross-session chance decoding accuracy was obtained by decoding the value differences using the neural activity from a different behavior session as suggested previously^45^. Using the neural activity of each session, we decoded the value differences from randomly selected 100 behavior sessions of different mice. The value differences of each randomly selected session were circularly permuted at a random trial number before decoding to randomize the first trial position in the reward probability blocks. Decoding results from the 100 sessions were averaged for the cross-session chance decoding accuracy.

### Coding axis similarity

We quantified the similarity of coding axes between paired sessions for OFC neurons and recurrent neurons in deep RL models. Our two-photon calcium imaging was performed at two different focus planes on alternating days for each mouse. Therefore, we quantified the similarity of the coding axis vectors from paired sessions that are 2 d apart. For each session pair, we first registered which neurons correspond to which in the paired sessions. Based on the cellular identity, we gave a unique ID to each shared neuron. Coding axis vector for each value-related signal on *k*th day was defined using the decoder weights from Eqs. (22–24) as follows:

25 $${\overrightarrow{{{\boldsymbol{c}}}^{{\boldsymbol{k}}}}}=\left[{\beta }_{1}^{k},{\beta }_{2}^{k},\ldots ,{\beta }_{n}^{k}\right]$$

where the numbers on the lower right (1, 2, …, *n*) indicate the unique IDs given to *n* neurons shared between paired imaging sessions. Cosine similarity of the coding axis vectors between *k*th day and (*k* + 2)th day is given by

26 $$\cos (\theta )=\frac{\overrightarrow{{{\boldsymbol{c}}}^{{\boldsymbol{k}}}}\cdot {\overrightarrow{{c}^{{\boldsymbol{k}}+{\bf{2}}}}}}{{\Big\Vert\, \overrightarrow{{{\boldsymbol{c}}}^{{\boldsymbol{k}}}}}\Big\Vert \Big\Vert\, {\overrightarrow{{{\boldsymbol{c}}}^{{\boldsymbol{k}}+{\bf{2}}}}}\Big\Vert }$$

where $\theta$ is the angle between the paired axis vectors in degrees and ||·|| denotes L2-norm of a vector.

For deep RL models, we similarly defined coding axis vectors using decoder weights from all 100 recurrent neurons. Cosine similarity was calculated for immediately adjacent sessions (*k*th session and (*k* + 1)th session).

### Relationships between value coding in neural activity and behavioral action policy

We analyzed the relationships between value coding in neural activity and behavioral action policy. We examined both their magnitude relationships and stability relationships. Because action values are updated based on reward history, we focused on the reward-based action policy axis (Eq. 13). The magnitude of behavioral reward history dependence was defined as follows using the weights from Eq. (13):

27 $${\mathrm {Reward}}\,{\mathrm {history}}\,{\mathrm {dependence}}=\mathop{\sum }\limits_{i=1}^{5}\left|{\beta }_{{{\mathrm{Rew}}C}\left(t-i\right)}^{\,k}\right|$$

where $\left|\bullet \right|$ denotes absolute value of the regression weight. The magnitude relationships between value coding and action policy were analyzed using this behavioral history dependence and the value decoding accuracy from neural population activity.

To analyze the across-session stability relationships between value coding and action policy, we calculated the angles of a coding axis pair and a policy axis pair between *k*th and (*k* + 2)th sessions as follows:

28 $${\theta }_{c}=\arccos \left(\frac{{\overrightarrow{{{\boldsymbol{c}}}^{{\boldsymbol{k}}}}}\cdot {\overrightarrow{{{\boldsymbol{c}}}^{{\boldsymbol{k}}+{\bf{2}}}}}}{\Big\Vert\, {\overrightarrow{{{\boldsymbol{c}}}^{{\boldsymbol{k}}}}}\Big\Vert \Big\Vert\, {\overrightarrow{{{\boldsymbol{c}}}^{{\boldsymbol{k}}+{\bf{2}}}}}\Big\Vert }\right)$$

29 $${\theta }_{p}=\arccos\left(\frac{{\overrightarrow{{{\boldsymbol{p}}}_{{\boldsymbol{r}}}^{{\boldsymbol{k}}}}}\cdot {\overrightarrow{{{\boldsymbol{p}}}_{{\boldsymbol{r}}}^{{\boldsymbol{k}}+{\bf{2}}}}}}{\Big\Vert\, {\overrightarrow{{{\boldsymbol{p}}}_{{\boldsymbol{r}}}^{{\boldsymbol{k}}}}}\Big\Vert \Big\Vert\, {\overrightarrow{{{\boldsymbol{p}}}_{{\boldsymbol{r}}}^{{\boldsymbol{k}}+{\bf{2}}}}}\Big\Vert }\right)\,$$

We used these angles instead of their cosines because cosine function introduces nonlinearity, which can skew the data distribution on a scatterplot.

### Mixed-effects models for statistics

We used mixed-effects models for the statistical analyses of nested data. lmer function in the lme4 *pa*ckage^71^ for parametric test or aligned rank transform (ART)^72^ for nonparametric test was used in R. ART was used for all statistical tests for the comparisons of regression weight sizes (including action bias size) because the weight distributions were severely skewed. lmer was used for the other statistical tests with mixed-effects models. The models used in this manuscript are as follows:

30 $$y \sim {\mathrm {session}}+\left(1|{\mathrm {subject}}\right)$$

where the fixed effect is the training session number and a random intercept is for the subject. This model was used to assess the action policy changes during training in Fig. 1i,l.

31 $$y \sim {\mathrm {opt}}+\left(0+{\mathrm {opt}}|{\mathrm {subject}}\right)+\left(1|{\mathrm {session}}\right)$$

where the fixed effect ${\mathrm {opt}}$ is 1 on inactivation trials and 0 on control trials, a random slope is for ${\mathrm {subject}}$ (mouse or deep RL) and a random intercept is for ${\mathrm {session}}$. This model was used for the paired comparisons in Figs. 3b, 5 and 6 and Extended Data Figs. 4i–m and 8.

32 $$y \sim {\mathrm {virus}}+\left(1|{\mathrm {day}}\right)$$

where the fixed effect ${\mathrm {virus}}$ is 1 for EGFP-paAIP2 mice and 0 on control EGFP mice and a random intercept is for training day (session numbers). This model was used for Fig. 2g,h,j and Extended Data Fig. 4d–h.

33 $$y \sim {\mathrm {virus}}+\left(1|{\mathrm {trial}}\right)$$

where the fixed effect ${\mathrm {virus}}$ is 1 for EGFP-paAIP2 mice and 0 for control EGFP mice and a random intercept is for the trial number from the probability block transition. This model was used for Extended Data Fig. 4a–c.

34 $$y \sim x+\left(1|{\mathrm {population}}\right)$$

where the random intercept is for simultaneously imaged neural population, and the fixed effect *x* and the observation *y* are the shuffling (0, not shuffled; 1, shuffled) and the decoding accuracy (Fig. 4d and Extended Data Fig. 6), the session number and the decoding accuracy (Fig. 7b and Extended Data Fig. 9a), the decoding accuracy of a value-related signal from OFC population activity and the behavioral history dependence (Fig. 7c and Extended Data Fig. 9b), the session pair number and the cosine similarity between value coding axes from the paired session (Fig. 7d and Extended Data Fig. 9c), the angle between value coding axes and the angle between policy axes (Fig. 7e and Extended Data Figs. 9d and 10), respectively.

### Statistics and reproducibility

#### Sample size

No statistics were used to predetermine the sample size.

#### Data exclusions

We excluded animals that did not learn the task either due to loss of motivation or sickness. For two-photon calcium imaging, we excluded neurons that were not consistently within the field-of-view during each imaging session.

#### Randomization

We allocated male mice from the same littermates randomly to paAIP2 group and control group for in vivo OFC plasticity suppression experiments. Selections of animals in the other experiments were completely random, and mice from different litters were mixed.

#### Blinding

For the experiments where we assessed the effects of OFC plasticity suppression on the learning curves, the type of virus injected (EGFP-P2ApaAIP2 or EGFP) was blinded to the trainer of mice. Data collection and analysis were not performed blind to the conditions of the other experiments.

### Data analysis software and library

We used Python3, MATLAB and R for data processing and analyses. All statistical tests are two-sided unless otherwise noted. Confidence interval (CI) and s.e.m were obtained by bootstrapping 1,000 times unless otherwise noted. lme4 package^71^ and ARTool^72^ were used in R for mixed effects models. We used TensorFlow2 (ref. ^73^) for training artificial neural networks and scikit-learn^67^ for training the other machine learning models. SciPy^74^ and Numpy^75^ were also used for numerical computations. Matplotlib^76^ and seaborn^77^ were used for data visualizations.

### Reporting summary

Further information on research design is available in the Nature Portfolio Reporting Summary linked to this article.

## Online content

Any methods, additional references, Nature Portfolio reporting summaries, source data, extended data, supplementary information, acknowledgements, peer review information; details of author contributions and competing interests; and statements of data and code availability are available at 10.1038/s41593-023-01485-3.

### Supplementary information

Supplementary Information Supplementary Fig. 1.

Reporting Summary

### Source data

Source Data Fig. 1 Numerical source data files for figures.

Source Data Fig. 2 Numerical source data files for figures.

Source Data Fig. 3 Numerical source data files for figures.

Source Data Fig. 4 Numerical source data files for figures.

Source Data Fig. 5 Numerical source data files for figures.

Source Data Fig. 6 Numerical source data files for figures.

Source Data Fig. 7 Numerical source data files for figures.

Source Data Extended Data Fig. 1 Numerical source data files for figures.

Source Data Extended Data Fig. 2 Numerical source data files for figures.

Source Data Extended Data Fig. 4 Numerical source data files for figures.

Source Data Extended Data Fig. 5 Numerical source data files for figures.

Source Data Extended Data Fig. 6 Numerical source data files for figures.

Source Data Extended Data Fig. 7 Numerical source data files for figures.

Source Data Extended Data Fig. 8 Numerical source data files for figures.

Source Data Extended Data Fig. 9 Numerical source data files for figures.

Source Data Extended Data Fig. 10 Numerical source data files for figures.

## Data Availability

The mouse behavior data and neural activity data are available at 10.5281/zenodo.8378063. The other generated datasets are available from the corresponding author upon reasonable request. Source data are provided with this paper.

## Code availability

Code to train a deep RL model and analyze its behaviors is available on GitHub (https://github.com/ryhattori/MetaRL-PRL) or 10.5281/zenodo.8368718.
