# Author Correction: Meta-reinforcement learning via orbitofrontal cortex

**DOI:** 10.1038/s41593-024-01718-z

**Published:** 2024-07-02

**Authors:** Ryoma Hattori, Nathan G. Hedrick, Anant Jain, Shuqi Chen, Hanjia You, Mariko Hattori, Jun-Hyeok Choi, Byung Kook Lim, Ryohei Yasuda, Takaki Komiyama

**Affiliations:** 1 Department of Neurobiology, University of California San Diego, La Jolla, CA USA; 2 Center for Neural Circuits and Behavior, University of California San Diego, La Jolla, CA USA; 3 Department of Neurosciences, University of California San Diego, La Jolla, CA USA; 4 Halıcıoğlu Data Science Institute, University of California San Diego, La Jolla, CA USA; 5 Max Planck Florida Institute for Neuroscience, Jupiter, FL USA; 6 Present Address: Department of Neuroscience, The Herbert Wertheim UF Scripps Institute for Biomedical Innovation & Technology, University of Florida, Jupiter, FL USA

**Keywords:** Neural circuits, Reward, Cortex

Correction to: *Nature Neuroscience* 10.1038/s41593-023-01485-3, published online 13 November 2023

In the version of the article initially published, in the Methods, the “Surgery for two-photon imaging and optogenetics” and “Surgery for extracellular spike recording” sections included the coordinate targeted for ORC imaging and recording, “~2.45 mm lateral and ~2.6 mm anterior from bregma”, which has now been amended to read “~1.45 mm lateral and ~2.6 mm anterior from bregma” in the HTML and PDF versions of the article.

