# Supplementary material for: Meta-reinforcement learning via orbitofrontal cortex
Source: Nat Neurosci. 2023 Nov 13;26(12):2182–91. doi: 10.1038/s41593-023-01485-3 (PMC10689244; doi:10.1038/s41593-023-01485-3)
Supplement: Supplementary file 1 — Supplementary Fig. 1. [file 41593_2023_1485_MOESM1_ESM.pdf]

nature neuroscience

Article

https://doi.org/10.1038/s41593-023-01485-3

Meta-reinforcement learning via orbitofrontal cortex

In the format provided by the authors and unedited

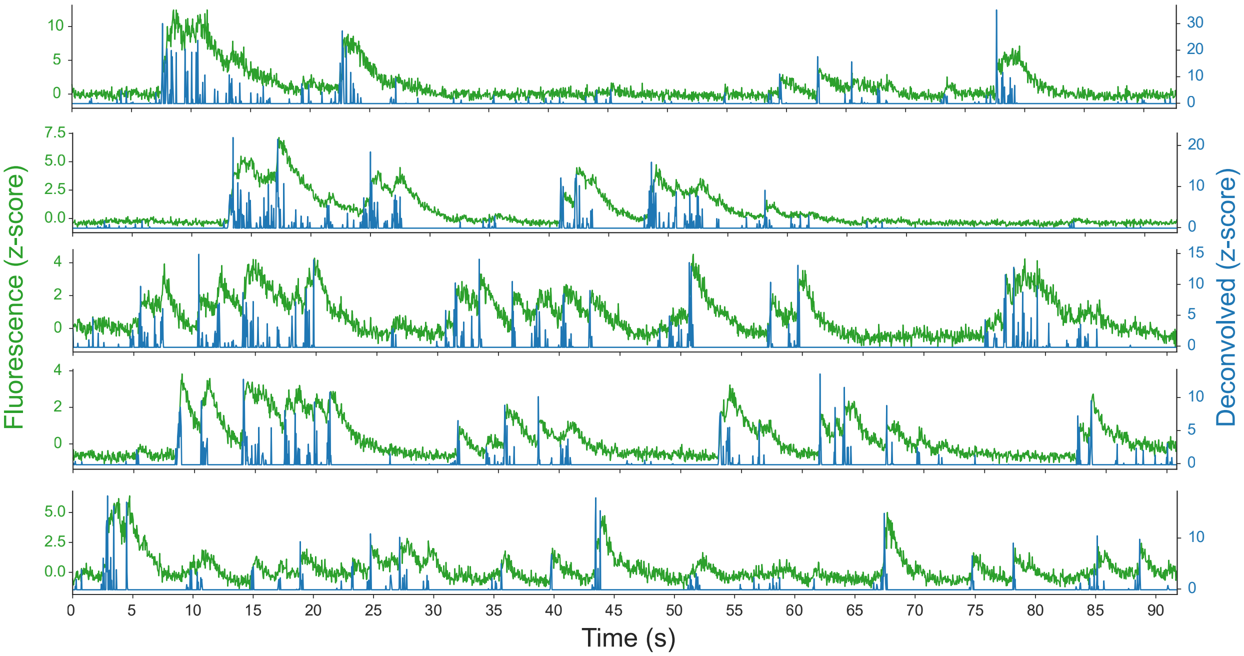

Supplementary Fig.1|GCaMP signals were deconvolved to estimate underlying spiking activity. Example deconvolutions of GCaMP fluorescence signals (green: raw fluorescence, blue: estimated spiking activity from deconvolution). Examples from 5 different neurons are shown. Deconvolutions were applied to estimate the underlying spiking activity of individual neurons. We used the estimated spiking activity for all neural activity analyses.
