# Supplementary material for: Meta-reinforcement learning via orbitofrontal cortex
Source: Nat Neurosci. 2023 Nov 13;26(12):2182–91. doi: 10.1038/s41593-023-01485-3 (PMC10689244; doi:10.1038/s41593-023-01485-3)
Supplement: Supplementary file 2 — Reporting Summary [file 41593_2023_1485_MOESM2_ESM.pdf]

nature portfolio

Corresponding author(s): Takaki Komiyama

Last updated by author(s): Sep 22, 2023

Reporting Summary

Nature Portfolio wishes to improve the reproducibility of the work that we publish. This form provides structure for consistency and transparency in reporting. For further information on Nature Portfolio policies, see our Editorial Policies and the Editorial Policy Checklist.

Statistics

For all statistical analyses, confirm that the following items are present in the figure legend, table legend, main text, or Methods section.

n/a	Confirmed	
☐	☒	The exact sample size (*n*) for each experimental group/condition, given as a discrete number and unit of measurement
☐	☒	A statement on whether measurements were taken from distinct samples or whether the same sample was measured repeatedly
☐	☒	The statistical test(s) used AND whether they are one- or two-sided<br>*Only common tests should be described solely by name; describe more complex techniques in the Methods section.*
☐	☒	A description of all covariates tested
☐	☒	A description of any assumptions or corrections, such as tests of normality and adjustment for multiple comparisons
☐	☒	A full description of the statistical parameters including central tendency (e.g. means) or other basic estimates (e.g. regression coefficient) AND variation (e.g. standard deviation) or associated estimates of uncertainty (e.g. confidence intervals)
☐	☒	For null hypothesis testing, the test statistic (e.g. *F*, *t*, *r*) with confidence intervals, effect sizes, degrees of freedom and *P* value noted<br>*Give P values as exact values whenever suitable.*
☒	☐	For Bayesian analysis, information on the choice of priors and Markov chain Monte Carlo settings
☐	☒	For hierarchical and complex designs, identification of the appropriate level for tests and full reporting of outcomes
☐	☒	Estimates of effect sizes (e.g. Cohen's *d*, Pearson's *r*), indicating how they were calculated

Our web collection on statistics for biologists contains articles on many of the points above.

Software and code

Policy information about availability of computer code

Data collection	ScanImage4.2 (Vidrio Technologies) for 2-photon imaging<br>Bpod v0.5 (JI Sanders, A Kepecs) and Bcontrol (C Brody) for behavior data collection
Data analysis	Python3 (TensorFlow2, scikit-learn, SciPy, Numpy), MATLAB, R (lme4, ARTool)

For manuscripts utilizing custom algorithms or software that are central to the research but not yet described in published literature, software must be made available to editors and reviewers. We strongly encourage code deposition in a community repository (e.g. GitHub). See the Nature Portfolio guidelines for submitting code & software for further information.

Data

Policy information about availability of data

All manuscripts must include a data availability statement. This statement should provide the following information, where applicable:

- Accession codes, unique identifiers, or web links for publicly available datasets
- A description of any restrictions on data availability
- For clinical datasets or third party data, please ensure that the statement adheres to our policy

The mouse behavior data and neural activity data were deposited to xxxx. The other generated datasets are available from the corresponding author upon reasonable request. Source data are provided with this paper.

Field-specific reporting

Please select the one below that is the best fit for your research. If you are not sure, read the appropriate sections before making your selection.

☒ Life sciences ☐ Behavioural & social sciences ☐ Ecological, evolutionary & environmental sciences

For a reference copy of the document with all sections, see nature.com/documents/nr-reporting-summary-flat.pdf

Life sciences study design

All studies must disclose on these points even when the disclosure is negative.

Sample size

No statistics were used to predetermine sample size. However, the sample sizes were chosen based on common practice in the field. We confirmed that the selected sample sizes had sufficient statistical power.

Data exclusions

We excluded animals that did not learn the task either due to loss of motivation or sickness. For 2-photon calcium imaging, we excluded neurons that were not consistently within the field-of-view during each imaging session.

Replication

All experiments and simulations were replicated as follows;
- We trained multiple artificial networks in this study (5 independently trained networks) from randomly initialized network weights.
- 14 distinct OFC neural populations for 2-photon imaging (7 mice, 2 focal planes per mouse).
- Each OFC population was imaged multiple times over training days (total number of 390 imaging sessions from 14 populations).
- 5 mice for each condition of paAIP2 experiments.
- The following number of mice and sessions were collected for inactivation experiments with different conditions. [ChrimsonR-tdTomato, ITI + Ready, bilateral (6 mice, 43 sessions)], [ChrimsonR-tdTomato, 2 sec ITI, bilateral (9 mice, 60 sessions)], [ChrimsonR-tdTomato, 5 sec ITI, bilateral (8 mice, 47 sessions)], [ChrimsonR-tdTomato, Ready, bilateral (10 mice, 62 sessions)]. [tdTomato, ITI + Ready, bilateral (5 mice, 30 sessions)], [tdTomato, 2 sec ITI, bilateral (8 mice, 49 sessions)], [tdTomato, 5 sec ITI, bilateral (8 mice, 46 sessions)], [tdTomato, Ready, bilateral (8 mice, 49 sessions)], [ChrimsonR-tdTomato, ITI + Ready, unilateral (4 mice, 177 sessions)]
- 16 spines from 8 neurons were used to assess the paAIP2 effects on spine enlargement in OFC neurons.
- Patch-clamp recording of OFC neurons were performed from 13 and 15 neurons before and after paAIP2 photostimulations.
- in vivo paAIP2 effects on spine plasticity were assessed using 4 mice with EGFP-P2A-paAIP2 expressions and 4 mice with only EGFP expressions.

Randomization

We allocated male mice from the same littermates randomly to paAIP2 group and control group for in vivo OFC plasticity suppression experiments. Selections of animals in the other experiments were completely random, and mice from different litters were mixed.

Blinding

For the experiments where we assessed the effects of OFC plasticity suppression on the learning curves, the type of virus injected (EGFP-P2A-paAIP2 or EGFP) was blinded to the trainer of mice. Data collection and analysis were not performed blind to the conditions of the other experiments.

Reporting for specific materials, systems and methods

We require information from authors about some types of materials, experimental systems and methods used in many studies. Here, indicate whether each material, system or method listed is relevant to your study. If you are not sure if a list item applies to your research, read the appropriate section before selecting a response.

Materials & experimental systems

n/a	Involved in the study	
☒	☐	Antibodies
☒	☐	Eukaryotic cell lines
☒	☐	Palaeontology and archaeology
☐	☒	Animals and other organisms
☒	☐	Human research participants
☒	☐	Clinical data
☒	☐	Dual use research of concern

Methods

n/a	Involved in the study	
☒	☐	ChIP-seq
☒	☐	Flow cytometry
☒	☐	MRI-based neuroimaging

Animals and other organisms

Policy information about studies involving animals; ARRIVE guidelines recommended for reporting animal research

Laboratory animals

We used adult mice (> 2 months of age) for most experiments except for the preparation of OFC slice cultures (4- to 6-day-old). The housing condition was 68-72°F temperature and 0-100% humidity.
C57BL/6 strain (Charles River) for wild-type.
CaMKIIa-tTA: B6;CBA-Tg(Camk2a-tTA)1Mmay/J [JAX 003010] for calcium imaging.
tetO-GCaMP6s:B6;DBA-Tg(tetO-GCaMP6s)2Niell/J [JAX 024742] for calcium imaging.
PV-Cre: B6;129P2-Pvalbtm1(cre)Arbr/J [JAX 008069] for inactivation experiments.

Both males and females are used for most experiments, but only male wild-type mice were used for experiments that examined the paAIP2 effects on mouse behaviors.

Wild animals	No wild animals were used in this study.
Field-collected samples	No field collected samples were used in the study.
Ethics oversight	All procedures were in accordance with the Institutional Animal Care and Use Committee at University of California San Diego

Note that full information on the approval of the study protocol must also be provided in the manuscript.
